# The modified beta transmuted family of distributions with applications using the exponential distribution

**DOI:** 10.1371/journal.pone.0258512

**Published:** 2021-11-18

**Authors:** Phillip Oluwatobi Awodutire, Oluwafemi Samson Balogun, Akintayo Kehinde Olapade, Ethelbert Chinaka Nduka

**Affiliations:** 1 Department of Mathematics and Computer Science, University of Africa, Toru Orua, Bayelsa State, Nigeria; 2 School of Computing, University of Eastern Finland, Kuopio, Finland; 3 Department of Mathematics, Obafemi Awolowo University, Ife, Osun State, Nigeria; 4 Department of Mathematics and Statistics, University of Port Harcourt, Port Harcourt, Rivers State, Nigeria; Universita degli Studi di Catania, ITALY

## Abstract

In this work, a new family of distributions, which extends the Beta transmuted family, was obtained, called the Modified Beta Transmuted Family of distribution. This derived family has the Beta Family of Distribution and the Transmuted family of distribution as subfamilies. The Modified beta transmuted frechet, modified beta transmuted exponential, modified beta transmuted gompertz and modified beta transmuted lindley were obtained as special cases. The analytical expressions were studied for some statistical properties of the derived family of distribution which includes the moments, moments generating function and order statistics. The estimates of the parameters of the family were obtained using the maximum likelihood estimation method. Using the exponential distribution as a baseline for the family distribution, the resulting distribution (modified beta transmuted exponential distribution) was studied and its properties. The modified beta transmuted exponential distribution was applied to a real life time data to assess its flexibility in which the results shows a better fit when compared to some competitive models.

## 1 Introduction

Due to complexity in distributions of real life data, there is need for developing distributions that are more flexible in fitting these data. The flexible distributions can be derived by addition of new parameters to the baseline distributions. Over years, many family of distributions has been developed. Examples like Beta-G [1], Weibull-G [2], Beta-Weibull-G [3], Modified Beta- G [4], Cubic Transmuted -G [5], Gompertz-G [6], Odd Lindley-G [7] e.t.c. Through these families of distributions, several models have been developed and applied to real life situations. [8] derived the transmuted-G family of distribution. In their work, they considered a baseline cumulative distribution function (cdf) G(x;*γ*) with corresponding probability density function (pdf) g(x;*γ*) and obtained the c.d.f of transmuted-G family of distribution P(x;*γ*) as

$$\begin{array}{c} I(x;\gamma )=G(x;\gamma )[1+\phi -\phi G(x;\gamma )] \end{array}$$ (1)

with the probability distribution function p.d.f as

$$\begin{array}{c} i(x;\gamma )=g(x;\gamma )[1+\phi -2\phi G(x;\gamma )] \end{array}$$ (2)

where *ϕ* is the transmuted parameter. When *ϕ* = 0 in Eqs 1 and 2, gives the p.d.f and the c.d.f of the baseline distribution.

In this work, a new family of distribution was derived that will be more flexible than the transmuted-G family of distribution by the addition of three more parameters to the transmuted-G family of distribution [8]. This concept is inspired by the work of Nadarajah et al. (2014), who obtained the modified beta-G families of distributions. This study will derive another family of distributions called the modified beta transmuted family of distributions which is more flexible and model fitting than that of Nadarajah et.al.(2014). Another important and crucial motivation is the study of modeling and analyses of lifetime data. The fitness of the assumed lifetime distribution, on the other hand, has a significant impact on the quality of statistical analyses. In a bid to achieve this, the modified beta- G family of distribution [4] was used to obtain the modified beta transmuted family of distribution. Given the c.d.f of baseline distribution G(x;*γ*), the c.d.f of the modified beta-G family A(x;*γ*) of distribution is given as

$$\begin{array}{c} A\left(x;\gamma \right)=\int_{0}^{\frac{\tau (G(x;\gamma ))}{1+((\tau -1)G(x;\gamma ))}}t^{\mu -1}{\left(1-t\right)}^{\sigma -1}dt \end{array}$$ (3)

which equivalently gives

$$\begin{array}{c} A\left(x;\gamma \right)=I_{\frac{\tau (G(x;\gamma ))}{1+((\tau -1)G(x;\gamma ))}}\left(\mu ,\sigma \right)=\frac{B(r;\mu ,\sigma )}{B(a,b)} \end{array}$$ (4)

and the corresponding p.d.f as

$$\begin{array}{c} a\left(x;\gamma \right)=\frac{\tau^{\mu }[G\left(x;\gamma \right){\left(G\left(x;\gamma \right)\right)}^{\mu -1}{\left(1-G\left(x;\gamma \right)\right)}^{b-1}]}{B\left(\mu ,\sigma \right)[1-(1-\tau )G(x;\gamma ){]}^{\mu +\sigma }} \end{array}$$ (5)

where $r=\frac{\tau (G(x;\gamma ))}{1+((\tau -1)G(x;\gamma ))}$ and *B*(*r*; *μ*, *σ*) is an incomplete beta function. where *μ* and *σ* are shape parameters, $I_{\frac{\tau G(x;\gamma )}{1+((\tau -1)G(x;\gamma ))}}\left(a,b\right)$ is the incomplete beta function ratio. If *μ* = *σ* = *τ* = 1, it gives the g(x;*γ*) and G(x;*γ*) of baseline distribution. Therefore, in the section 2, the new family of distribution was derived. In Section 3, the mixture representation of the p.d.f and the c.d.f of the family of distribution was obtained, section 4 studied the statistical properties and the estimation of parameters of the family of distribution. Then, in Section 5, the family of distribution was studied using the exponential distribution as the baseline distribution. The properties were studied and applied to a real data to assess its performance when compared to some sub-models. Section 6 gives the conclusion of the work.

## 2 Derivation of the Modified Beta Transmuted-G (MBTG) family of distribution

Incorporating Eqs 1 in 3, the c.d.f of the MBTG family of distribution is derived as

$$\begin{array}{c} A\left(x;\gamma \right)=\int_{0}^{\frac{\tau G(x;\gamma )[1+\phi -\phi G(x;\gamma )]}{1+((\tau -1)G(x;\gamma )[1+\phi -\phi G(x;\gamma )])}}t^{\mu -1}{\left(1-t\right)}^{\sigma -1}dt \end{array}$$ (6)

which gives

$$\begin{array}{c} A\left(x;\gamma \right)=I_{\frac{\tau G(x;\gamma )[1+\phi -\phi G(x;\gamma )]}{1+((\tau -1)\tau G(x;\gamma )[1+\phi -\phi G(x;\gamma )])}}\left(\mu ,\sigma \right)=\frac{B(f;\mu ,\sigma )}{B(a,b)} \end{array}$$ (7)

where $f=\frac{\tau G(x;\gamma )[1+\phi -\phi G(x;\gamma )]}{1+((\tau -1)\tau G(x;\gamma )[1+\phi -\phi G(x;\gamma )])}$.

From Eq 6, the p.d.f of the MBTG family of distribution is obtained as

$$\begin{array}{c} a\left(x;\gamma \right)=\frac{\tau^{\mu }[g\left(x;\gamma \right)\left[1+\phi -2\phi G\left(x;\gamma \right)\right]{\left(G(x;\gamma )[1+\phi -\phi G(x;\gamma )]\right)}^{\mu -1}{\left(1-G(x;\gamma )[1+\phi -\phi G(x;\gamma )]\right)}^{b-1}]}{B\left(\mu ,\sigma \right)[1-(1-\tau )G(x;\gamma )[1+\phi -\phi G(x;\gamma )]{]}^{\mu +\sigma }} \end{array}$$ (8)

where *μ*,*σ* and *τ* are the shape parameters and *ϕ* is the transmuted parameter. The MBTG family of distribution has the following as the submodels;

1. when *τ* = 1, the beta transmuted-G family of distribution [9] is obtained
2. when *τ* = *μ* = *σ* = 1, the MBTG family of distribution becomes the Transmuted-G family [8]
3. when *τ* = 1 and *ϕ* = 0, it gives the Beta-G family [1]
4. when *τ* = *μ* = *σ* = 1 and *ϕ* = 0, it gives the baseline distribution G(x;*γ*)
5. when *τ*,*σ* = 1 it gives the Exponentiated Transmuted G family [10]

The survival function s(x;*γ*) of MBTG family of distribution is obtained as

$$\begin{array}{c} s\left(x;\gamma \right)=1-\frac{B(f;\mu ,\sigma )}{B(\mu ,\sigma )}=\frac{B(\mu ,\sigma )-B(f;\mu ,\sigma )}{B(\mu ,\sigma )} \end{array}$$ (9)

and the hazard function h(x;*γ*) is obtained as

$$\begin{array}{c} h\left(x;\gamma \right)=\frac{B\left(\mu ,\sigma \right)\tau^{\mu }[g\left(x;\gamma \right)\left[1+\phi -2\phi G\left(x;\gamma \right)\right]{\left(G(x;\gamma )[1+\phi -\phi G(x;\gamma )]\right)}^{\mu -1}{\left(1-G(x;\gamma )[1+\phi -\phi G(x;\gamma )]\right)}^{b-1}]}{\left(B\left(\mu ,\sigma \right)-B\left(f;\mu ,\sigma \right)\right)(1-(1-\tau )G(x;\gamma )[1+\phi -\phi G(x;\gamma )]{)}^{\mu +\sigma }} \end{array}$$ (10)

### 2.1 Sub-models of the MBTG family of distributions

In this section, three special models of the MBTG family of distribution is presented. These models generalize some models that are already existing in literatures. The models have baselines of Gompertz (G), Exponential(E) and Lindley(L) distributions.

### 2.2 Modified Beta Transmuted Gompertz (MBTGo) distribution

The pdf and cdf of Gompertz distribution are given as

$$\begin{array}{c} g\left(x;\tau ,\epsilon \right)=\epsilon \tau e^{\left(\tau +\epsilon x-\tau e^{\epsilon x}\right)} \end{array}$$

and

$$\begin{array}{c} G\left(x;\tau ,\epsilon \right)=1-e^{\left(-\tau \left(e^{\frac{x}{\epsilon }}-1\right)\right)} \end{array}$$

respectively, for x >0 and *τ*, *ϵ* > 0. Now, the pdf *f*_*MBTGo*_ and hazard function *h*_*MBTGo*_ of the MBTGo distribution is given as

$$\begin{array}{c} f_{MBTGo}=\frac{\tau^{\mu }[\left(\epsilon \tau e^{\left(\tau +\epsilon x-\tau e^{\epsilon x}\right)}\right)\left[1+\phi -2\phi \left(1-e^{\left(-\tau \left(e^{\frac{x}{\epsilon }}-1\right)\right)}\right)\right]{\left(\left(1-e^{\left(-\tau \left(e^{\frac{x}{\epsilon }}-1\right)\right)}\right)\left[1+\phi -\phi \left(1-e^{\left(-\tau \left(e^{\frac{x}{\epsilon }}-1\right)\right)}\right)\right]\right)}^{\mu -1}]}{B\left(\mu ,\sigma \right){\left[1-\left(1-\tau \right)\left(1-e^{\left(-\tau \left(e^{\frac{x}{\epsilon }}-1\right)\right)}\right)\left[1+\phi -\phi \left(1-e^{\left(-\tau \left(e^{\frac{x}{\epsilon }}-1\right)\right)}\right)\right]\right]}^{\mu +\sigma }} \end{array}$$

$$\begin{array}{c} (1-\left(1-e^{\left(-\tau \left(e^{\frac{x}{\epsilon }}-1\right)\right)}\right)\left[1+\phi -\phi \left(1-e^{\left(-\tau \left(e^{\frac{x}{\epsilon }}-1\right)\right)}\right)\right]{)}^{\sigma -1} \end{array}$$

and

$$\begin{array}{c} h_{MBTGo}=\frac{B\left(\mu ,\sigma \right)\tau^{\mu }[\left(\frac{\tau e^{\frac{x}{\epsilon }}}{\epsilon }e^{\left(-\tau \left(e^{\frac{x}{\epsilon }}-1\right)\right)}\right)\left[1+\phi -2\phi \left(1-e^{\left(-\tau \left(e^{\frac{x}{\epsilon }}-1\right)\right)}\right)\right]]}{\left(B\left(\mu ,\sigma \right)-B\left(f;\mu ,\sigma \right)\right)(1-\left(1-\tau \right)G\left(x;\gamma \right)[1+\phi -\phi \left(1-e^{\left(-\tau \left(e^{\frac{x}{\epsilon }}-1\right)\right)}\right)]{)}^{\mu +\sigma }} \end{array}$$

$$\begin{array}{c} {\left(\left(1-e^{\left(-\tau \left(e^{\frac{x}{\epsilon }}-1\right)\right)}\right)\left[1+\phi -\phi \left(1-e^{\left(-\tau \left(e^{\frac{x}{\epsilon }}-1\right)\right)}\right)\right]\right)}^{\mu -1}{\left(1-\left(1-e^{\left(-\tau \left(e^{\frac{x}{\epsilon }}-1\right)\right)}\right)\left[1+\phi -\phi \left(1-e^{\left(-\tau \left(e^{\frac{x}{\epsilon }}-1\right)\right)}\right)\right]\right)}^{\sigma -1} \end{array}$$

**Fig 1:**
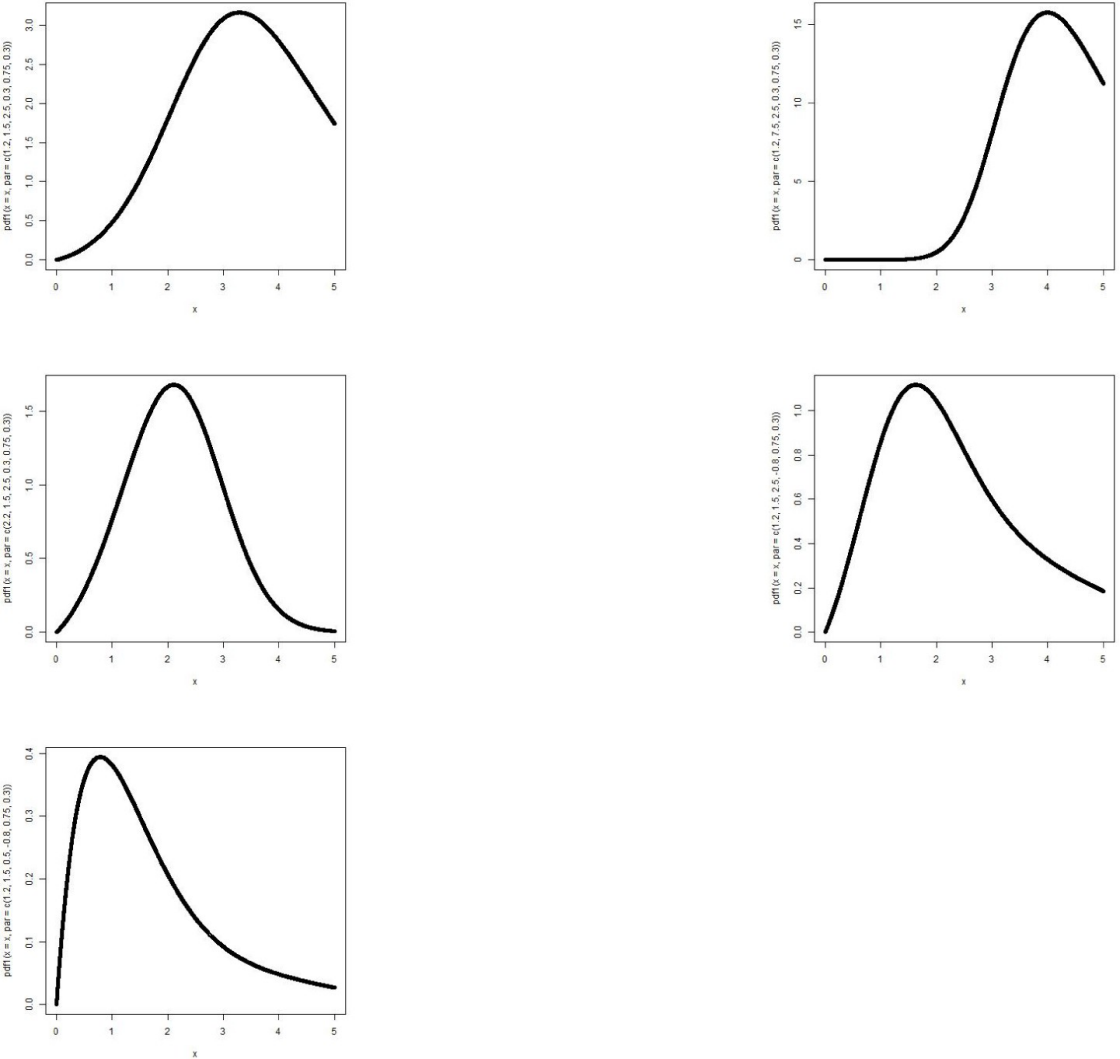
Graphs of p.d.f of MBTGo with various parameter values.

**Fig 2:**
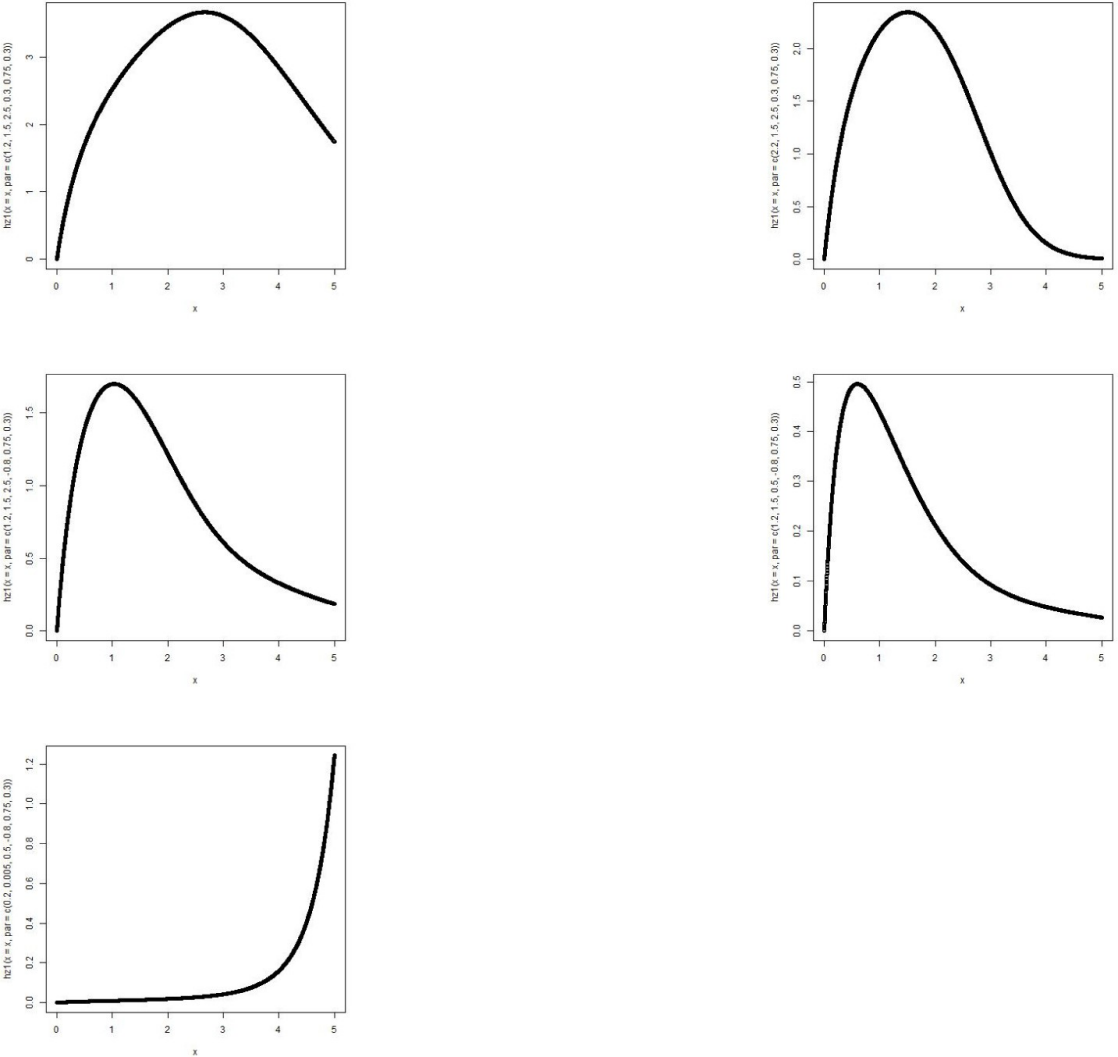
Graphs of hazard function of MBTGo with various parameter values.

The MBTGo distribution includes the Transmuted Gompertz(TG) [11] when *θ* = *ζ* = *ϕ* = 1. For *θ* = *α* = *ρ* = 1, the MBTGo becomes Beta Gompertz(BGo) distribution [12]. For *θ* = *ζ* = 1, MBTGo reduces to Exponentiated Transmuted Gompertz(ETGo) distribution (**NEW**). Plots of the density function and the hazard function of the MBTGo with various assigned parameter values are shown in Figs 1 and 2 respectively.

#### 2.2.1 Modified Beta Transmuted Exponential (MBTE) distribution

The pdf and cdf of exponential distribution are given as

$$\begin{array}{c} g\left(x;\beta \right)=\beta e^{-\beta x} \end{array}$$

$$\begin{array}{c} G\left(x;\beta \right)=1-e^{-\beta x} \end{array}$$

Therefore, the pdf (*f*_*MBTE*_) and hazard function (*h*_*MBTE*_) of the MBTE distribution is given as

$$\begin{array}{c} f_{MBTEx}=\frac{\tau^{\mu }[\left(\beta e^{-\beta x}\right)\left[1+\phi -2\phi \left(1-e^{-\beta x}\right)\right]{\left(\left(1-e^{-\beta x}\right)\left[1+\phi -\phi \left(1-e^{-\beta x}\right)\right]\right)}^{\mu -1}]}{B\left(\mu ,\sigma \right)[1-\left(1-\tau \right)\left(1-e^{-\beta x}\right)\left[1+\phi -\phi \left(1-e^{-\beta x}\right)\right]{]}^{\mu +\sigma }} \end{array}$$

$$\begin{array}{c} (1-\left(1-e^{-\beta x}\right)\left[1+\phi -\phi \left(1-e^{-\beta x}\right)\right]{)}^{b-1} \end{array}$$

and

$$\begin{array}{c} h_{MBTE}=\frac{B\left(\mu ,\sigma \right)\tau^{\mu }[\left(\beta e^{-\beta x}\right)\left[1+\phi -2\phi \left(1-e^{-\beta x}\right)\right]{\left(\left(1-e^{-\beta x}\right)\left[1+\phi -\phi \left(1-e^{-\beta x}\right)\right]\right)}^{\mu -1}]}{\left(B\left(\mu ,\sigma \right)-B\left(f;\mu ,\sigma \right)\right){\left(1-\left(1-\tau \right)\left(1-e^{-\beta x}\right)\left[1+\phi -\phi \left(1-e^{-\beta x}\right)\right]\right)}^{\mu +\sigma }} \end{array}$$

$$\begin{array}{c} (1-\left(1-e^{-\beta x}\right)\left[1+\phi -\phi \left(1-e^{-\beta x}\right)\right]{)}^{\sigma -1} \end{array}$$

**Fig 3:**
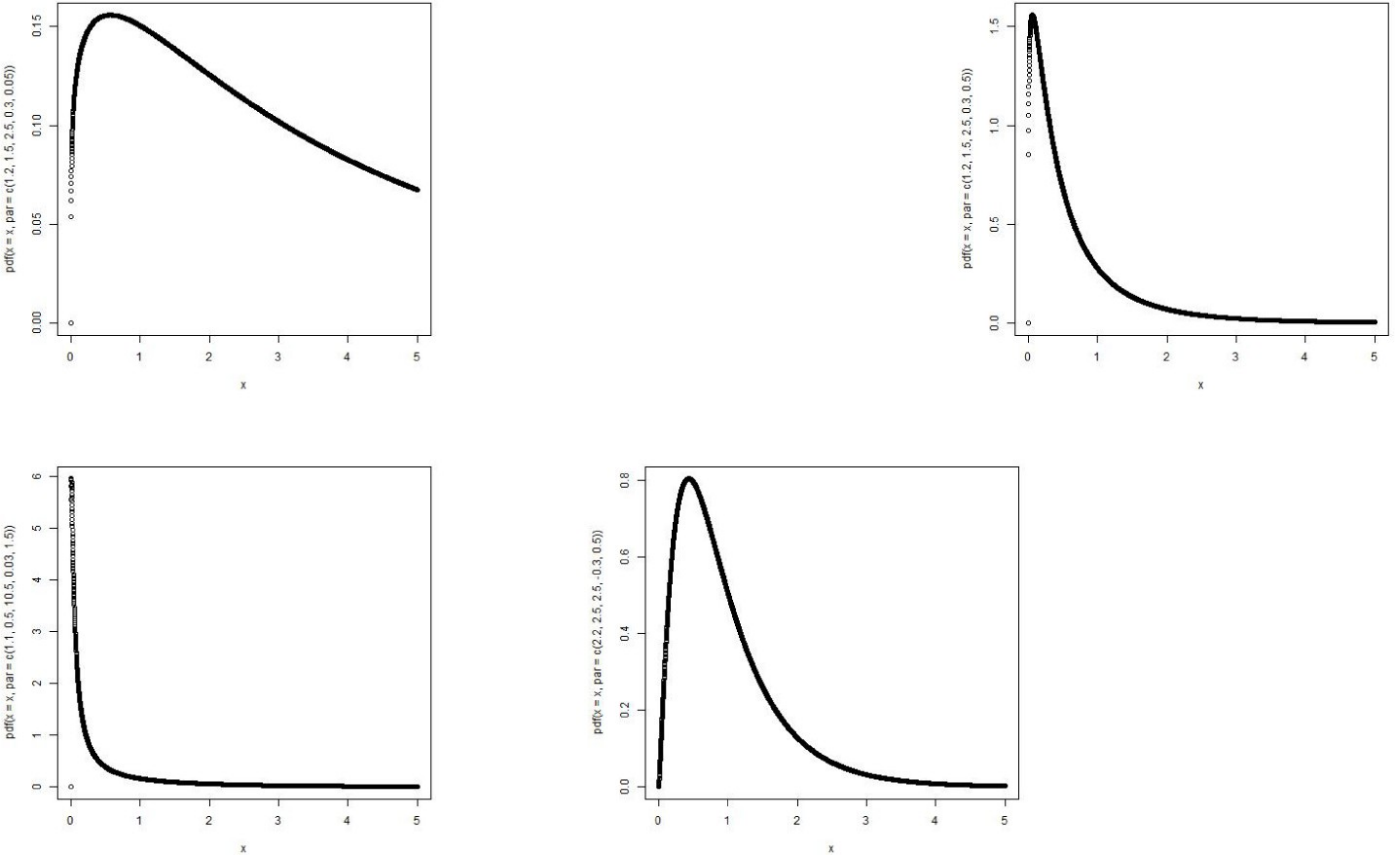
Graphs of p.d.f of MBTED with various parameter values.

**Fig 4:**
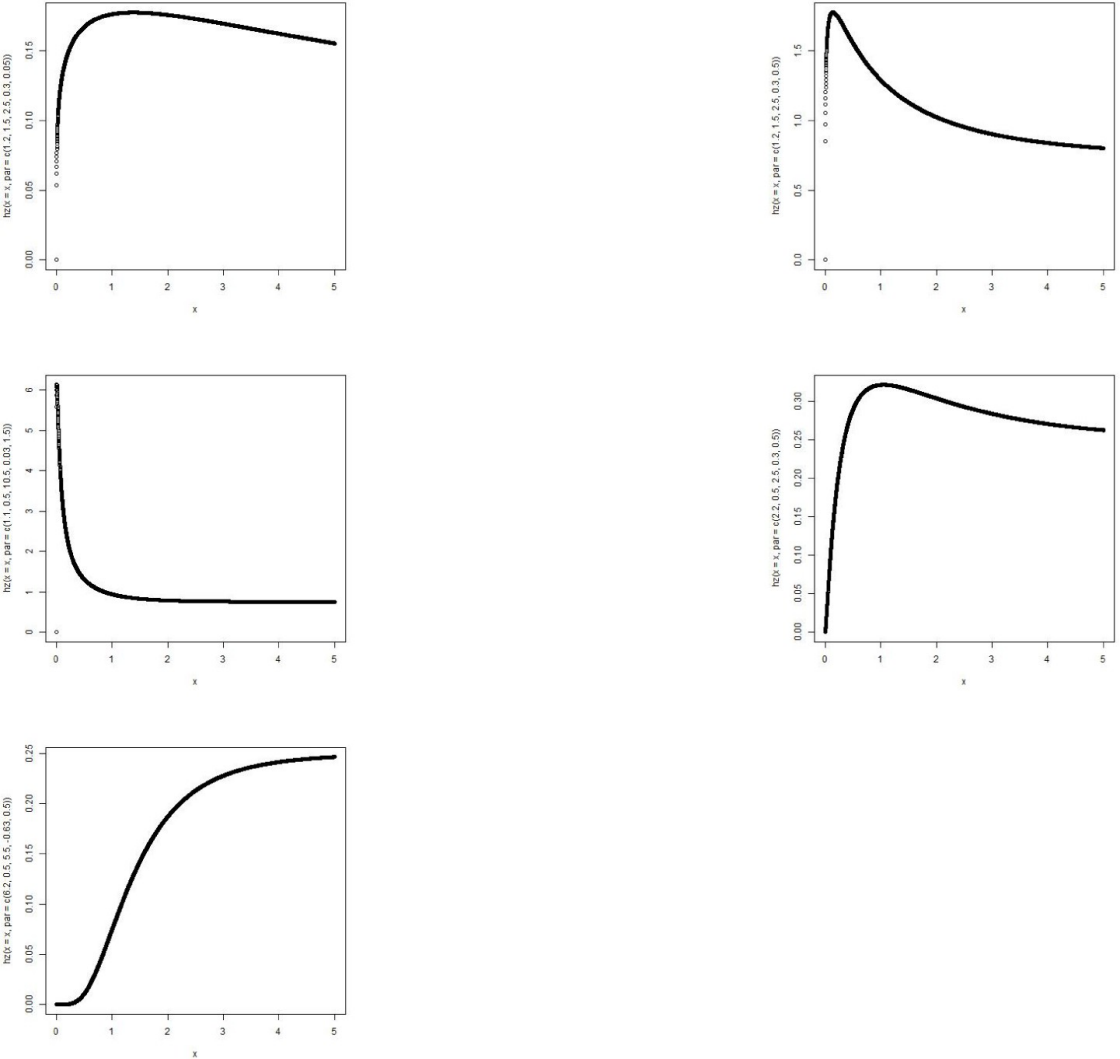
Graphs of hazard function of MBTED with various parameter values.

The MBTE distribution includes the Transmuted Exponential [13] when *θ* = *ζ* = *ϕ* = 1. For *θ* = *α* = *ρ* = 1, the MBTE distribution becomes Beta Exponential(BE) distribution [14]. For *θ* = *ζ* = 1, MBTE reduces to Exponentiated Transmuted Exponential(ETE) distribution [15]. Plots of the density function and the hazard function of the MBTE distribution with various assigned parameter values are shown in Figs 3 and 4.

### 2.3 Modified Beta Transmuted Lindley (MBTL) distribution

The pdf and cdf of lindley distribution are given as

$$\begin{array}{c} G\left(x;\beta ,\lambda \right)=1-\frac{e^{-\beta x}\left(1+\beta +\beta x\right)}{1+\beta } \end{array}$$

$$\begin{array}{c} g\left(x;\beta ,\lambda \right)=\frac{\beta^{2}}{\beta +1}\left(1+x\right)e^{-\beta x} \end{array}$$

Now, the pdf *f*_*MBTL*_ and hazard function *h*_*MBTL*_ MBTL distribution is given as

$$\begin{array}{c} f_{MBTL}=\frac{\tau^{\mu }[\left(\frac{\beta^{2}}{\beta +1}\left(1+x\right)e^{-\beta x}\right)\left[1+\phi -2\phi \left(1-\frac{e^{-\beta x}\left(1+\beta +\beta x\right)}{1+\beta }\right)\right]{\left(\left(1-\frac{e^{-\beta x}\left(1+\beta +\beta x\right)}{1+\beta }\right)\left[1+\phi -\phi \left(1-\frac{e^{-\beta x}\left(1+\beta +\beta x\right)}{1+\beta }\right)\right]\right)}^{\mu -1}]}{B\left(\mu ,\sigma \right)[1-\left(1-\tau \right)\left(1-\frac{e^{-\beta x}\left(1+\beta +\beta x\right)}{1+\beta }\right)\left[1+\phi -\phi \left(1-\frac{e^{-\beta x}\left(1+\beta +\beta x\right)}{1+\beta }\right)\right]{]}^{\mu +\sigma }} \end{array}$$

$$\begin{array}{c} (1-\left(1-\frac{e^{-\beta x}\left(1+\beta +\beta x\right)}{1+\beta }\right)\left[1+\phi -\phi \left(1-\frac{e^{-\beta x}\left(1+\beta +\beta x\right)}{1+\beta }\right)\right]{)}^{b-1} \end{array}$$

and

$$\begin{array}{c} h_{MBTL}=\frac{B\left(\mu ,\sigma \right)\tau^{\mu }[\left(\frac{\beta^{2}}{\beta +1}\left(1+x\right)e^{-\beta x}\right)\left[1+\phi -2\phi \left(1-\frac{e^{-\beta x}\left(1+\beta +\beta x\right)}{1+\beta }\right)\right]]}{\left(B\left(\mu ,\sigma \right)-B\left(f;\mu ,\sigma \right)\right){\left(1-\left(1-\tau \right)\left(1-\frac{e^{-\beta x}\left(1+\beta +\beta x\right)}{1+\beta }\right)\left[1+\phi -\phi \left(1-\frac{e^{-\beta x}\left(1+\beta +\beta x\right)}{1+\beta }\right)\right]\right)}^{\mu +\sigma }} \end{array}$$

$$\begin{array}{c} (\left(1-\frac{e^{-\beta x}\left(1+\beta +\beta x\right)}{1+\beta }\right)\left[1+\phi -\phi \left(1-\frac{e^{-\beta x}\left(1+\beta +\beta x\right)}{1+\beta }\right)\right]{)}^{\mu -1} \end{array}$$

$$\begin{array}{c} (1-\left(1-\frac{e^{-\beta x}\left(1+\beta +\beta x\right)}{1+\beta }\right)\left[1+\phi -\phi \left(1-\frac{e^{-\beta x}\left(1+\beta +\beta x\right)}{1+\beta }\right)\right]{)}^{\sigma -1} \end{array}$$

**Fig 5:**
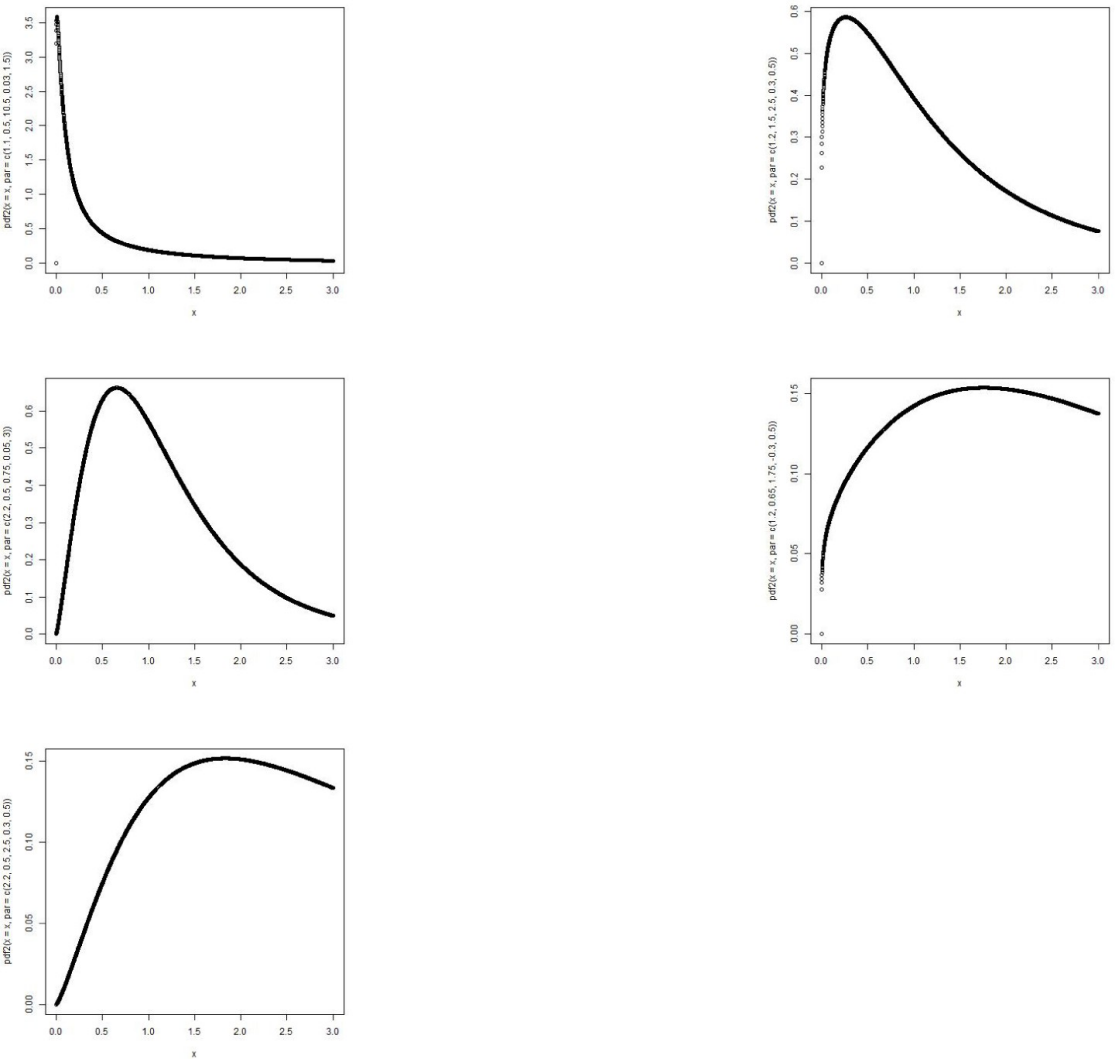
Graphs of p.d.f of MBTL with various parameter values.

**Fig 6:**
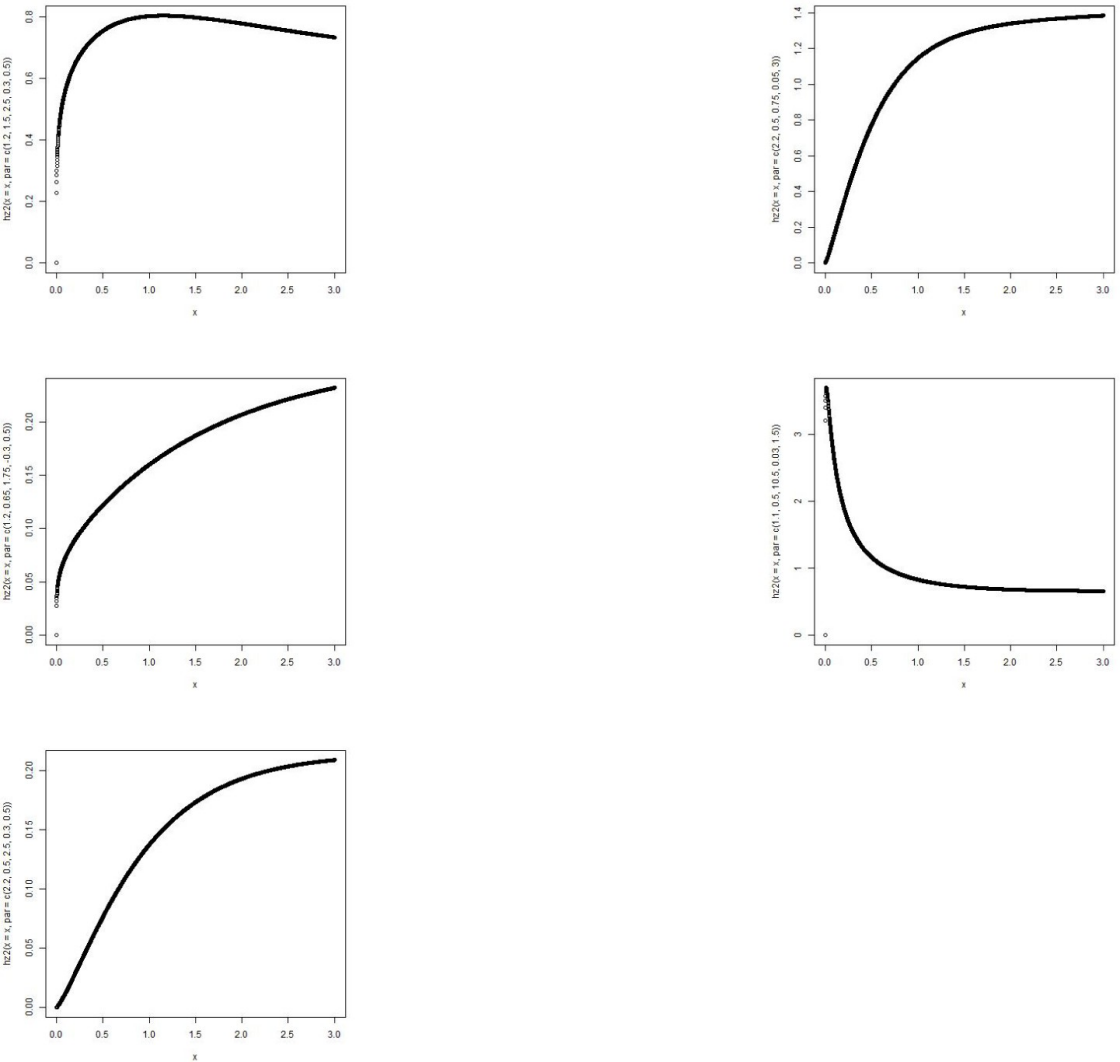
Graphs of hazard function of MBTL with various parameter values.

The MBTL distribution includes the Transmuted Lindley(TL) [16] when *θ* = *ζ* = *ϕ* = 1. For *θ* = *α* = *ρ* = 1, the MBTL becomes Beta Lindley(BL) distribution [17]. For *θ* = *ζ* = 1, MBTL reduces to Exponentiated Transmuted Lindey(ETL) distribution [18]. Plots of the density function and the hazard function of the MBTL with various assigned parameter values are shown in Figs 5 and 6.

From the plots of the submodels of the MBTG distribution, it shows that the proposed family of distribution can be rightly skewed, symmetric, reverse J shape and other forms of shape inferring that this family of distribution will be suitable in modeling different form of real life situations due to its flexibility.

## 3 Mixture representation

In this section, the mixture representation of the p.d.f of the MBTG family of distribution is derived. Having this expression simplifies the derivation of some statistical properties of MBTG family.

Using the binomial expression, as written in Wolfram Statistics

$$\begin{array}{c} {\left(1-z\right)}^{q-1}=\sum_{k=0}^{\infty }{\left(-1\right)}^{k}\left(\begin{array}{c} q\\ k \end{array}\right)z^{k} \end{array}$$ (11)

such that |*z*| < 1 and *k* > 0 real non-integer.

From Eq 8, Considering

$$\begin{array}{c} E=[1-(1-\tau )G(x;\gamma )[1+\phi -\phi G(x;\gamma )]{]}^{\mu +\sigma } \end{array}$$ (12)

By the application of the binomial expression, Eq 12 is

$$\begin{array}{c} E=\sum_{k=0}^{\infty }\left(\begin{array}{c} -\mu -\sigma \\ k \end{array}\right){\left(-1\right)}^{k}{\left(1-\tau \right)}^{k}{\left(G\left(x;\gamma \right)\left[1+\phi -\phi G\left(x;\gamma \right)\right]\right)}^{k} \end{array}$$ (13)

Likewise considering

$$\begin{array}{c} W=[1-G(x;\gamma )[1+\phi -\phi G(x;\gamma )]{]}^{\sigma -1} \end{array}$$ (14)

and using the binomial expression, Eq 14 is

$$\begin{array}{c} W=\sum_{l=0}^{\infty }\left(\begin{array}{c} \sigma -1\\ l \end{array}\right){\left(-1\right)}^{l}{\left(G\left(x;\gamma \right)\left[1+\phi -\phi G\left(x;\gamma \right)\right]\right)}^{l} \end{array}$$ (15)

Applying Eqs 13 and 15 to Eq 8, the mixture representation of the p.d.f of the MBTG family is

$$\begin{array}{c} a\left(x;\gamma \right)=\sum_{k=0}^{\infty }\sum_{l=0}^{\infty }\begin{array}{c} \left(\begin{array}{c} -\mu -\sigma \\ k \end{array})(\begin{array}{c} \sigma -1\\ l \end{array}\right) \end{array}{\left(-1\right)}^{k+l}{\left(1-\tau \right)}^{k}g\left(x;\gamma \right)\left[1+\phi -2\phi G\left(x;\gamma \right)\right]{\left(G\left(x;\gamma \right)\left[1+\phi -\phi G\left(x;\gamma \right)\right]\right)}^{\mu +k+l-1} \end{array}$$ (16)

Furthermore, Eq 16 can written in form of the exponentiated transmuted G as

$$\begin{array}{c} a\left(x;\gamma \right)=\sum_{k=0}^{\infty }\sum_{l=0}^{\infty }g_{r}\Pi_{\mu +k+l} \end{array}$$ (17)

where

$$\begin{array}{c} g_{r}=\begin{array}{c} \left(\begin{array}{c} -\mu -\sigma \\ k \end{array})(\begin{array}{c} \sigma -1\\ l \end{array}\right) \end{array}{\left(-1\right)}^{k+l}{\left(1-\tau \right)}^{k}{\left(\mu +k+l\right)}^{-1} \end{array}$$

and

$$\begin{array}{c} \Pi_{\mu +k+l}=\left(\mu +k+l\right)[g(x;\gamma )[1+\phi -2\phi G(x;\gamma )](G(x;\gamma )[1+\phi -\phi G(x;\gamma ){]}^{\mu +k+l-1} \end{array}$$

Π_*μ*+*k*+*l*_ is the p.d.f of the exponentiated transmuted-G family of distribution with index parameters *μ*+k+l.

From Eq 17, the corresponding c.d.f of the MBTG family of distribution is

$$\begin{array}{c} A\left(x;\gamma \right)=\sum_{k=0}^{\infty }\sum_{l=0}^{\infty }g_{r}\beta_{\mu +k+l} \end{array}$$ (18)

*β*_*μ*+*k*+*l*_ is the c.d.f of the exponentiated transmuted-G family of distribution with index parameters *μ*+k+l.

## 4 Statistical properties

In this section, some statistical properties of the MBTG family of distribution are studied. The properties include order statistics, moments, moment generating function, shanon entropy and the quantile function.

### 4.1 Order statistics

Order statistics make their appearance in many areas of statistical theory and practice. Let *X*, *X*_2_, *X*_3_, *X*_4_, …, *X*_*n*_ be random sample generated from the MBTG family of distributions. The p.d.f of *i*^*th*^ order statistic, *X*_*i*:*n*_, can be written as

$$\begin{array}{c} f_{\left(i:n\right)}\left(x;\gamma \right)=n\left(\begin{array}{l} n-1\\ n-j \end{array}\right)f\left(x;\gamma \right){\left[1-F\left(x;\gamma \right)\right]}^{i-1}F{\left(x;\gamma \right)}^{n-i} \end{array}$$ (19)

Inserting Eqs 8, and 6 in 19, then

$$\begin{array}{c} a_{\left(i:n\right)}\left(x;\gamma \right)=n\left(\begin{array}{l} n-1\\ n-j \end{array}\right)[\frac{\tau^{\mu }[G\left(x;\gamma \right){\left(G\left(x;\gamma \right)\right)}^{\mu -1}{\left(1-G\left(x;\gamma \right)\right)}^{b-1}]}{B\left(\mu ,\sigma \right)[1-(1-\tau )G(x;\gamma ){]}^{\mu +\sigma }}][\frac{B(\mu ,\sigma )-B(f;\mu ,\sigma )}{B(\mu ,\sigma )}{]}^{j-1}[\frac{B(f;\mu ,\sigma )}{B(\mu ,\sigma )}{]}^{n-j} \end{array}$$ (20)

The first order statistics *X*(1) has the marginal p.d.f. obtained as

$$\begin{array}{c} n[\frac{\tau^{\mu }[G\left(x;\gamma \right){\left(G\left(x;\gamma \right)\right)}^{\mu -1}{\left(1-G\left(x;\gamma \right)\right)}^{b-1}]}{B\left(\mu ,\sigma \right)[1-(1-\tau )G(x;\gamma ){]}^{\mu +\sigma }}][\frac{B(f;\mu ,\sigma )}{B(\mu ,\sigma )}{]}^{n-1} \end{array}$$ (21)

while the last order statistics have the marginal p.d.f as

$$\begin{array}{c} n[\frac{\tau^{\mu }[G\left(x;\gamma \right){\left(G\left(x;\gamma \right)\right)}^{\mu -1}{\left(1-G\left(x;\gamma \right)\right)}^{b-1}]}{B\left(\mu ,\sigma \right)[1-(1-\tau )G(x;\gamma ){]}^{\mu +\sigma }}][\frac{B(\mu ,\sigma )-B(f;\mu ,\sigma )}{B(\mu ,\sigma )}{]}^{n-1} \end{array}$$ (22)

In terms of the mixture representation, order statistics of the MBTG family of distribution is

$$\begin{array}{c} a_{\left(i:n\right)}\left(x;\gamma \right)=n\left(\begin{array}{l} n-1\\ n-j \end{array}\right)[\sum_{k=0}^{\infty }\sum_{l=0}^{\infty }g_{r}\Pi_{\mu +k+l}][\sum_{k=0}^{\infty }\sum_{l=0}^{\infty }g_{r}\beta_{\mu +k+l}{]}^{n-i}[1-(\sum_{k=0}^{\infty }\sum_{l=0}^{\infty }g_{r}\beta_{\mu +k+l}){]}^{i-1} \end{array}$$ (23)

and the first order marginal p.d.f and last order marginal p.d.f given as

$$\begin{array}{c} n\left(\begin{array}{l} n-1\\ n-j \end{array}\right)[\sum_{k=0}^{\infty }\sum_{l=0}^{\infty }g_{r}\Pi_{\mu +k+l}][\sum_{k=0}^{\infty }\sum_{l=0}^{\infty }g_{r}\beta_{\mu +k+l}{]}^{n-1} \end{array}$$ (24)

$$\begin{array}{c} n\left(\begin{array}{l} n-1\\ n-j \end{array}\right)[\sum_{k=0}^{\infty }\sum_{l=0}^{\infty }g_{r}\Pi_{\mu +k+l}][1-(\sum_{k=0}^{\infty }\sum_{l=0}^{\infty }g_{r}\beta_{\mu +k+l}){]}^{n-1} \end{array}$$ (25)

### 4.2 Moments

The *r*^*th*^ moment of X, say $\psi_{r}^{\prime }$ follows from Eq 17 as

$$\begin{array}{c} \psi_{r}^{\prime }=E\left[X^{r}\right]=\sum_{k=0}^{\infty }\sum_{l=0}^{\infty }g_{r}E\left[\Pi_{\mu +k+l}^{r}\right] \end{array}$$ (26)

Therefore $E[\Pi_{\mu +k+l}^{r}]$ is the r-th moment of the exp-Transmuted G family.

The *n*^*th*^ central moment of X, say *M*_*n*_ is given by

$$\begin{array}{c} M_{n}=E{\left[X-\psi_{1}^{\prime }\right]}^{n}=\sum_{r=0}^{n}\left(\begin{array}{c} n\\ r \end{array}\right){\left(\psi_{1}^{\prime }\right)}^{n-r}E\left[X^{r}\right] \end{array}$$ (27)

$$\begin{array}{c} \sum_{r=0}^{n}\sum_{k=0}^{\infty }{\left(-1\right)}^{n-r}g_{r}\left(\begin{array}{c} n\\ r \end{array}\right){\left(\psi_{1}^{\prime }\right)}^{n-r}E\left[\Pi_{\mu +k+l}^{r}\right] \end{array}$$ (28)

### 4.3 Moment generating function

Using the expression as in Eq 17, the moment generating function of the MBTG family of distribution is

$$\begin{array}{c} M_{x}\left(t\right)=\sum_{k=0}^{\infty }\sum_{l=0}^{\infty }g_{r}M_{\mu +k+l}\left(t\right) \end{array}$$ (29)

where *M*_*μ*+*k*+*l*_(*t*) is the moment generating function of the exp-Transmuted G family of distribution.

### 4.4 Quantile function

The quantile function of the distribution is discussed here. If X MBTG(*μ*, *σ*, *τ*, *ϕ*, *γ*), then the quantile function of X can be simulated as

$$\begin{array}{c} X=G^{-1}(\frac{\phi +1-\sqrt{{\left(\phi +1\right)}^{2}+4\phi R}}{2\phi };\gamma ) \end{array}$$ (30)

where

$$\begin{array}{c} R=\frac{I_{U}^{-1}\left(\mu ,\sigma \right)}{c-(I_{U}^{-1}\left(\mu ,\sigma \right)\left(c-1\right))} \end{array}$$

$I_{u}^{-1}\left(\mu ,\sigma \right)$ gives B(*μ*,*σ*) and U ∼ univariate[0, 1].

### 4.5 Parameter estimation

Several approaches for parameter estimation exist in the literature but maximum likelihood method is the most commonly employed. The maximum likelihood estimators (MLEs) enjoy desirable properties and can be used when constructing confidence intervals and also in test statistics. The normal approximation for these estimators in large sample theory is easily handled either analytically or numerically. So, estimation of the unknown parameters for the MBTG family are determined only by maximum likelihood estimation method. Here, the MLEs of the parameters for complete samples only. Given samples *X*_1_, *X*_2_, …, *X*_*n*_ that follows the MBTG family of distribution, then the loglikelihood function l is given as

$$\begin{array}{c} l=n\mu \;\text{ln}\;\tau -n\;\text{ln}\;B\left(\mu ,\sigma \right)+n\;\text{ln}\;g\left(x;\gamma \right)+\sum_{i=1}^{n}\;\text{ln}\;\left(1+\phi -2\phi G\left(x;\gamma \right)\right)+\left(\mu -1\right)\sum_{i=1}^{n}\;\text{ln}\;\left(G\left(x_{i}\right)\left(1+\phi -\phi G\left(x;\gamma \right)\right)\right) \end{array}$$

$$\begin{array}{c} +\left(\sigma -1\right)\sum_{i=1}^{n}\;\text{ln}\;\left(1-G\left(x_{i}\right)\left(1+\phi -\phi G\left(x;\gamma \right)\right)\right)-\left(\mu +\sigma \right)\sum_{i=1}^{n}\;\text{ln}\;\left(1-(\left(1-\tau \right)G\left(x_{i}\right)\left(1+\phi -\phi G\left(x;\gamma \right)\right))\right) \end{array}$$ (31)

Differentiating Eq 31 with the respective distribution parameters, to have

$$\begin{array}{c} \frac{\partial l}{\partial \mu }=n\;\text{ln}\;\tau --n(\frac{\Gamma^{\prime }\left(\mu \right)}{\Gamma (\mu )}-\frac{\Gamma^{\prime }\left(\mu +\sigma \right)}{\Gamma (\mu +\sigma )})+\sum_{i=1}^{n}\;\text{ln}\;\left(G\left(x_{i}\right)\left(1+\phi -\phi G\left(x;\gamma \right)\right)\right) \end{array}$$

$$\begin{array}{c} -\sum_{i=1}^{n}\;\text{ln}\;\left(1-(\left(1-\tau \right)G\left(x_{i}\right)\left(1+\phi -\phi G\left(x;\gamma \right)\right))\right) \end{array}$$ (32)

$$\begin{array}{c} \frac{\partial l}{\partial \sigma }=-n(\frac{\Gamma^{\prime }\left(\sigma \right)}{\Gamma (\sigma )}-\frac{\Gamma^{\prime }\left(\mu +\sigma \right)}{\Gamma (\mu +\sigma )})+\sum_{i=1}^{n}\;\text{ln}\;\left(1-[G\left(x_{i}\right)\left(1+\phi -\phi G\left(x;\gamma \right)\right)]\right) \end{array}$$

$$\begin{array}{c} -\sum_{i=1}^{n}\;\text{ln}\;\left(1-(\left(1-\tau \right)G\left(x_{i}\right)\left(1+\phi -\phi G\left(x;\gamma \right)\right))\right) \end{array}$$ (33)

$$\begin{array}{c} \frac{\partial l}{\partial \tau }=\frac{n\mu }{\tau }-\left(a+b\right)\sum_{i=1}^{n}\frac{G\left(x_{i}\right)\left(1+\phi -\phi G\left(x;\gamma \right)\right)}{\;\text{ln}\;(1-(\left(1-\tau \right)G\left(x_{i}\right)\left(1+\phi -\phi G\left(x;\gamma \right)\right)))} \end{array}$$ (34)

$$\begin{array}{c} \frac{\partial l}{\partial \phi }=\sum_{i=1}^{n}\frac{1-2G(x;\gamma )}{1+\phi -2\phi G(x;\gamma )}+\left(\mu -1\right)\sum_{i=1}^{n}\frac{G(x;\gamma )(1-G(x;\gamma ))}{G(x;\gamma )(1+\phi -\phi (G(x;\gamma )))}-\left(\sigma -1\right)\sum_{i=1}^{n}\frac{G(x;\gamma )(1-G(x;\gamma ))}{1-G(x;\gamma )(1+\phi -\phi (G(x;\gamma )))} \end{array}$$

$$\begin{array}{c} +\left(\mu +\sigma \right)\sum_{i=1}^{n}\frac{\left(1-\tau )G(x;\gamma )(1-G(x;\gamma )\right)}{1-((1-\tau )G(x;\gamma )(1+\phi -\phi (G(x;\gamma ))))} \end{array}$$ (35)

$$\begin{array}{c} \frac{\partial l}{\partial \gamma }=\frac{ng^{\prime }\left(x;\gamma \right)}{g(x;\gamma )}+\sum_{i=1}^{n}\frac{-2\phi G^{\prime }\left(x;\gamma \right)}{1+\phi -2\phi G(x;\gamma )}+\left(\mu -1\right)\sum_{i=1}^{n}\frac{G^{\prime }\left(x;\gamma \right)\left(1+\phi -2\phi G\left(x;\gamma \right)\right)}{G(x;\gamma )(1+\phi -\phi (G(x;\gamma )))} \end{array}$$

$$\begin{array}{c} -\left(\sigma -1\right)\sum_{i=1}^{n}\frac{G^{\prime }\left(x;\gamma \right)\left(1+\phi -2\phi G\left(x;\gamma \right)\right)}{1-(G(x;\gamma )(1+\phi -\phi (G(x;\gamma ))))}+\left(a+b\right)\sum_{i=1}^{n}\frac{\left(1-\tau \right)G^{\prime }\left(x;\gamma \right)\left[1+\phi -2\phi G\left(x;\gamma \right)\right]}{1-((1-\tau )G(x;\gamma )(1+\phi -\phi (G(x;\gamma ))))} \end{array}$$ (36)

Setting the set of Eqs in 32, 33, 34, 35, 36 to be equals to zero and solving them simultaneously yields the MLE $\hat{\delta }$ = ($\hat{\zeta },\hat{\phi }$,$\hat{\theta }$,$\hat{\alpha }$,$\hat{\rho }$,$\hat{\gamma }$) of *δ* = (*ζ*,*ϕ*,*θ*,*α*,*ρ*,*γ*). Solving these equations cannot be done analytically. This can be achieved by the aid of statistical software using iterative methods such as Newton-Raphson type algorithms to solve numerically.

For interval estimation of the model parameters, the observed information matrix is required. For interval estimation and test of hypothesis on the parameters (*ζ*, *ϕ*, *θ*, *α*, *ρ*, *θ*), to obtain a 6x6 unit information matrix

$$J=[\begin{array}{cccccc} J_{\zeta ,\zeta } & J_{\zeta ,\phi } & J_{\zeta ,\theta } & J_{\zeta ,\alpha } & J_{\zeta ,\rho } & J_{\zeta ,\gamma }\\ J_{\zeta ,\phi } & J_{\phi ,\phi } & J_{\phi ,\theta } & J_{\phi ,\alpha } & J_{\phi ,\rho } & J_{\phi ,\gamma }\\ J_{\zeta ,\theta } & J_{\phi ,\theta } & J_{\theta ,\theta } & J_{\theta ,\alpha } & J_{\theta ,\rho } & J_{\theta ,\gamma }\\ J_{\zeta ,\alpha } & J_{\alpha ,\phi } & J_{\alpha ,\theta } & J_{\alpha ,\alpha } & J_{\alpha ,\rho } & J_{\alpha ,\gamma }\\ J_{\zeta ,\rho } & J_{\rho ,\phi } & J_{\rho ,\theta } & J_{\rho ,\alpha } & J_{\rho ,\rho } & J_{\rho ,\gamma }\\ J_{\zeta ,\gamma } & J_{\gamma ,\phi } & J_{\gamma ,\theta } & J_{\gamma ,\alpha } & J_{\gamma ,\rho } & J_{\gamma ,\gamma } \end{array}]$$

The corresponding elements are derived by the second derivatives of *l* with respect to the parameters.

Under conditions that are fulfilled for parameters, the asymptotic distribution of $\sqrt{n}\left(\hat{\delta }-\delta \right)$ is $N_{6}\left(0,J{\left(\hat{\delta }\right)}^{-1}\right)$ distribution of *δ* can be used to construct approximate confidence intervals and confidence regions for the parameters and for the hazard and survival functions. The asymptotic normality is also useful for testing goodness of fit of the beta type I generalized half logistic distribution and for comparing this distribution with some of its special sub-models using one of these two well known asymptotically equivalent test statistics- namely, the likelihood ratio statistic and Wald statistic. An asymptotic confidence interval with significance level *τ* for each parameter *δ*_*i*_ is given by

$$\begin{array}{c} ACI\left(\delta_{i},100\left(1-\tau \right)\right)=\hat{\delta }-z_{\frac{\tau }{2}}\sqrt{J^{\hat{\delta },\hat{\delta }}},\delta +z_{\frac{\delta }{2}}\sqrt{J^{\hat{\delta },\hat{\delta }}} \end{array}$$ (37)

where $J^{\hat{\delta },\hat{\delta }}$ is the *i*^*t*^*h* diagonal element of $K_{n}{\left(\hat{\delta }\right)}^{-1}$ for *i* = 1, 2, 3, 4, 5, 6 and *z*_*τ*/2_ is the quantile of the standard normal distribution.

## 5 The modified beta transmuted exponential distribution

In this section the exponential distribution is considered as a baseline distribution of the MBTG family of distribution. The exponential has been studied and many generalizations have been made by different authors. Some of these works employed the use of transmutation approach to derived the generalization of the exponential distribution. Such works includes the transmuted exponential, exponentiated transmuted exponential, exponentiated cubic exponential e.t.c. The p.d.f of the exponential distribution is

$$\begin{array}{c} g\left(x;\lambda \right)=\lambda e^{-\lambda x}\;x>0,\lambda >0 \end{array}$$ (38)

with c.d.f as

$$\begin{array}{c} G\left(x;\lambda \right)=1-e^{-\lambda x} \end{array}$$ (39)

where λ is a scale parameter. Therefore inserting the Eq 38 into Eq 8, the p.d.f of the Modified Beta Transmuted Exponential Distribution *q*_*E*_(*x*; *γ*) is derived as

$$\begin{array}{c} q_{E}\left(x;\lambda \right)=\frac{\tau^{\mu }e^{-\lambda x}\left(1-\phi +2\phi e^{-\lambda x}\right){\left(1-e^{-\lambda x}+\phi e^{-\lambda x}-\phi e^{-2\lambda x}\right)}^{\mu -1}{\left(e^{-\lambda x}-\phi e^{-\lambda x}+\phi e^{-2\lambda x}\right)}^{\sigma -1}}{B\left(\mu ,\sigma \right)[1-\left(1-\tau \right)\left(1-e^{-\lambda x}+\phi e^{-\lambda x}-\phi e^{-2\lambda x}\right)]} \end{array}$$ (40)

and the c.d.f *Q*_*E*_(*x*; λ) as

$$\begin{array}{c} Q_{E}\left(x;\lambda \right)=I_{M(x;\lambda )}\left(\mu ,\sigma \right)=\frac{B(M(x;\lambda );\mu ,\sigma )}{B(\mu ,\sigma )} \end{array}$$ (41)

where $M\left(x;\lambda \right)=\frac{\tau \left(1-e^{-\lambda x}+\phi e^{-\lambda x}-\phi e^{-2\lambda x}\right)\left(1-\phi +2\phi e^{-2\lambda x}\right)}{1+(\left(\tau -1\right)\left(1-e^{-\lambda x}\right)\left(1+\phi e^{-\lambda x}\right))}$ and *B*(*M*(*x*; *γ*);*μ*, *σ*) is an incomplete beta function.

For the distribution, *x* > 0, λ, *τ*, *μ*, *σ*>0 and |*p*| < 1.

### 5.1 Mixture representation of the MBTED

In this subsection, the mixture representation of the MBTED is derived. This will help derive the analytical expression of the distribution and will be useful in obtaining some properties of the MBTED.

Inserting Eqs 38 and 39 in Eq 16, the mixture representation of the p.d.f of MBTED is obtained as

$$\begin{array}{c} q_{E}\left(x;\lambda \right)=\sum_{k=0}^{\infty }\sum_{l=0}^{\infty }\left(\begin{array}{c} -\mu -\sigma \\ k \end{array}\right)\left(\begin{array}{c} \sigma -1\\ l \end{array}\right){\left(-1\right)}^{k+l}{\left(1-\tau \right)}^{k}\lambda e^{-\lambda x}\left[1-\phi +2\phi e^{-\lambda x}\right] \end{array}$$

$$\begin{array}{c} {\left(\left(1-e^{-\lambda x}\right)\left[1+\phi e^{-\lambda x}\right]\right)}^{\mu +k+l-1} \end{array}$$ (42)

Re-writing Eq 42 in terms of the p.d.f of exp-transmuted exponential distribution, it gives

$$\begin{array}{c} q_{E}\left(x;\lambda \right)=\sum_{k=0}^{\infty }\sum_{l=0}^{\infty }g_{r}\chi_{\mu +k+l} \end{array}$$ (43)

where

$$\begin{array}{c} g_{r}=\left(\begin{array}{c} -\mu -\sigma \\ k \end{array}\right)\left(\begin{array}{c} \sigma -1\\ l \end{array}\right){\left(-1\right)}^{k+l}{\left(1-\tau \right)}^{k}{\left(\mu +k+l\right)}^{-1} \end{array}$$

and

$$\begin{array}{c} \chi_{\mu +k+l}=\left(\mu +k+l\right)\lambda e^{-\lambda x}\left[1-\phi +2\phi e^{-\lambda x}\right]{\left(\left(1-e^{-\lambda x}\right)\left[1+\phi e^{-\lambda x}\right]\right)}^{\mu +k+l-1} \end{array}$$

*χ*_*μ*+*k*+*l*_ is the p.d.f of the exponentiated transmuted exponential distribution with index parameters *μ*+k+l as derived by [15].

From Eq 43, the corresponding c.d.f of the MBTG family of distribution is

$$\begin{array}{c} Q_{E}\left(x;\lambda \right)=\sum_{k=0}^{\infty }\sum_{l=0}^{\infty }g_{r}\Theta_{\mu +k+l} \end{array}$$ (44)

Θ_*μ*+*k*+*l*_ = ((1 − *e*^−λ*x*^)[1 + *ϕe*^−λ*x*^])^*μ*+*k*+*l*^ is the c.d.f of the exponentiated transmuted exponential distribution with index parameters *μ*+k+l.

The survival function of the MBTED is

$$\begin{array}{c} s\left(x;\lambda \right)=1-I_{M(x;\gamma )}\left(a,b\right)=\frac{B(\mu ,\sigma )-B(M(x;\gamma );\mu ,\sigma )}{B(\mu ,\sigma )} \end{array}$$ (45)

and the hazard function as

$$\begin{array}{c} h\left(x;\lambda \right)=\frac{\tau^{\mu }e^{-\lambda x}\left(1-\phi +2\phi e^{-2\lambda x}\right){\left(1-e^{-\lambda x}+\phi e^{-\lambda x}-\phi e^{-2\lambda x}\right)}^{\mu -1}\left(e^{-\lambda x}-\phi e^{-\lambda x}+\phi e^{-2\lambda x}\right)}{\left(B\left(M\left(x;\lambda \right);\mu ,\sigma \right)\right)[1-\left(1-\tau \right)\left(1-e^{-\lambda x}+\phi e^{-\lambda x}-\phi e^{-2\lambda x}\right)]} \end{array}$$ (46)

### 5.2 Quantile function

Inverting *q*_*E*_(*x*; λ) = U, the quantile function of the MBTED is determined as

$$\begin{array}{c} x=\frac{-1}{\lambda }\;\text{ln}\;(1-(\frac{\phi +1-\sqrt{{\left(\phi +1\right)}^{2}+4\phi R}}{2\phi };\gamma )) \end{array}$$ (47)

where

$$\begin{array}{c} R=\frac{I_{U}^{-1}\left(\mu ,\sigma \right)}{c-(I_{U}^{-1}\left(\mu ,\sigma \right)\left(c-1\right))} \end{array}$$

$I_{u}^{-1}\left(\mu ,\sigma \right)$ gives B(*μ*,*σ*) and U ∼ univariate[0, 1].

### 5.3 Order statistics of MBTED

Let *X*_1_, *X*_2_, *X*_3_, *X*_4_, …, *X*_*n*_ be random sample generated from the MBTED distributions. The p.d.f of *i*^*th*^ order statistic, *X*_*i*:*n*_, can be written as

$$\begin{array}{c} q_{E}\left(i:n\right)\left(x;\lambda \right)=n\left(\begin{array}{c} n-1\\ n-j \end{array}\right)q_{E}\left(x;\lambda \right){\left[1-Q_{E}\left(x;\lambda \right)\right]}^{i-1}{\left(Q_{E}\left(x;\lambda \right)\right)}^{n-i} \end{array}$$ (48)

Inserting Eqs 40 and 41 in 48, the order statistics of the MBTED has the expression as

$$\begin{array}{c} q_{E}\left(i:n\right)\left(x;\lambda \right)=[\frac{\tau^{\mu }e^{-\lambda x}\left(1-\phi +2\phi e^{-\lambda x}\right){\left(1-e^{-\lambda x}+\phi e^{-\lambda x}-\phi e^{-2\lambda x}\right)}^{\mu -1}{\left(e^{-\lambda x}-\phi e^{-\lambda x}+\phi e^{-2\lambda x}\right)}^{\sigma -1}}{B\left(\mu ,\sigma \right)[1-\left(1-\tau \right)\left(1-e^{-\lambda x}+\phi e^{-\lambda x}-\phi e^{-2\lambda x}\right)]}] \end{array}$$

$$\begin{array}{c} {}[\frac{B(M(x;\gamma );\mu ,\sigma )}{B(\mu ,\sigma )}{]}^{i-1}[\frac{B(\mu ,\sigma )-B(M(x;\gamma );\mu ,\sigma )}{B(\mu ,\sigma )}{]}^{n-1} \end{array}$$ (49)

In terms of the mixture representation, order statistics of the MBTG family of distribution can be written as

$$\begin{array}{c} q_{E}\left(i:n\right)\left(x;\lambda \right)=n\left(\begin{array}{c} n-1\\ n-j \end{array}\right)[\sum_{k=0}^{\infty }\sum_{l=0}^{\infty }g_{r}\Pi_{\mu +k+l}][\sum_{k=0}^{\infty }\sum_{l=0}^{\infty }g_{r}\beta_{\mu +k+l}{]}^{n-i}[1-(\sum_{k=0}^{\infty }\sum_{l=0}^{\infty }g_{r}\beta_{\mu +k+l}){]}^{i-1} \end{array}$$ (50)

and the first order marginal p.d.f and last order marginal p.d.f given as

$$\begin{array}{c} n\left(\begin{array}{c} n-1\\ n-j \end{array}\right)[\sum_{k=0}^{\infty }\sum_{l=0}^{\infty }g_{r}\Pi_{\mu +k+l}][\sum_{k=0}^{\infty }\sum_{l=0}^{\infty }g_{r}\beta_{\mu +k+l}{]}^{n-1} \end{array}$$ (51)

$$\begin{array}{c} n\left(\begin{array}{c} n-1\\ n-j \end{array}\right)[\sum_{k=0}^{\infty }\sum_{l=0}^{\infty }g_{r}\Pi_{\mu +k+l}][1-(\sum_{k=0}^{\infty }\sum_{l=0}^{\infty }g_{r}\beta_{\mu +k+l}){]}^{n-1} \end{array}$$ (52)

### 5.4 Moments of MBTED

The moments of the Exponential Transmuted exponential distribution, as established by [15] is

$$\begin{array}{c} E\left[\Pi_{\mu +k+l}^{r}\right]=\left(\mu +k+l\right)\sum_{m=0}^{\infty }\sum_{w=0}^{\infty }\sum_{z=0}^{1}{\left(-1\right)}^{m}\rho^{w+z}{\left(1-\rho \right)}^{1-z}(\begin{array}{c} a+k+l-1\\ m \end{array})\left(\begin{array}{c} a+k+l-1\\ w \end{array}\right)\left(\begin{array}{c} 1\\ z \end{array}\right) \end{array}$$

$$\begin{array}{c} \frac{2^{z}\Gamma r+1}{{\left(i+j+m+1\right)}^{r+1}\rho^{r}} \end{array}$$ (53)

the moments of the MBTED is derived as

$$\begin{array}{c} E\left[X^{r}\right]=\sum_{k=0}^{\infty }\sum_{l=0}^{\infty }\sum_{m=0}^{\infty }\sum_{w=0}^{\infty }\sum_{z=0}^{1}{\left(-1\right)}^{m+k+l}{\left(1-\tau \right)}^{k}\rho^{w+z}{\left(1-\rho \right)}^{1-z} \end{array}$$

$$\begin{array}{c} (\begin{array}{c} a+k+l-1\\ m \end{array})(\begin{array}{c} a+k+l-1\\ w \end{array})(\begin{array}{c} 1\\ z \end{array})(\begin{array}{c} -\mu -\sigma \\ k \end{array})(\begin{array}{c} \sigma -1\\ l \end{array})\frac{2^{z}\Gamma (r+1)}{{\left(i+j+m+1\right)}^{r+1}\rho^{r}} \end{array}$$ (54)

From the expression in Eq 54, the mean E[X], second moment E[*X*^2^], Variance, Kurtosis and Skewness can be derived.

### 5.5 Moment generating function of MBTED

Using the moment generating function as established by [15], to have the moment generating function of MBTED as

$$\begin{array}{c} M_{X}\left(t\right)=\sum_{k=0}^{\infty }\sum_{l=0}^{\infty }\sum_{m=0}^{\infty }\sum_{w=0}^{\infty }\sum_{z=0}^{1}{\left(-1\right)}^{m+k+l}{\left(1-\tau \right)}^{k}\rho^{w+z}{\left(1-\rho \right)}^{1-z} \end{array}$$

$$(\begin{array}{c} a+k+l-1\\ m \end{array})(\begin{array}{c} a+k+l-1\\ w \end{array})(\begin{array}{c} 1\\ z \end{array})(\begin{array}{c} -\mu -\sigma \\ k \end{array})(\begin{array}{c} \sigma -1\\ l \end{array})\frac{(\mu +k+l)2^{z}\lambda }{\lambda (m+w+z+1)-t}$$ (55)

### 5.6 Shanon entropy

Entropy measures the uncertainty of a random variable X. The entropy of the MBTED is

$$\begin{array}{c} B=-E[log(f(x))] \end{array}$$ (56)

$$\begin{array}{c} B=-E[log\left(\frac{\tau^{\mu }e^{-\lambda x}\left(1-\phi +2\phi e^{-\lambda x}\right){\left(1-e^{-\lambda x}+\phi e^{-\lambda x}-\phi e^{-2\lambda x}\right)}^{\mu -1}{\left(e^{-\lambda x}-\phi e^{-\lambda x}+\phi e^{-2\lambda x}\right)}^{\sigma -1}}{B\left(\mu ,\sigma \right)[1-\left(1-\tau \right)\left(1-e^{-\lambda x}+\phi e^{-\lambda x}-\phi e^{-2\lambda x}\right)]}\right)] \end{array}$$ (57)

This can be estimated iteratively.

### 5.7 Parameter estimation of MBTED

If samples *X*_1_, *X*_2_, …, *X*_*n*_ is set of reandom samples distributed to the MBTED, then the loglikelihood function l is given as

$$\begin{array}{c} l=n\mu \;\text{ln}\;\tau -n\;\text{ln}\;B\left(\mu ,\sigma \right)+\sum_{i=1}^{n}\;\text{ln}\;\left(\lambda e^{-\lambda x}\right)+\sum_{i=1}^{n}\;\text{ln}\;\left(1-\phi +2\phi e^{-\lambda x}\right)+\left(\mu -1\right)\sum_{i=1}^{n}\;\text{ln}\;\left(1-e^{-\lambda x}+\lambda e^{-\lambda x}-\lambda e^{-2\lambda x}\right) \end{array}$$

$$\begin{array}{c} +\left(\sigma -1\right)\sum_{i=1}^{n}\;\text{ln}\;\left(e^{-\lambda x}-\lambda e^{-\lambda x}+\lambda e^{-2\lambda x}\right)-\left(\mu +\sigma \right)\sum_{i=1}^{n}\;\text{ln}\;\left(1-\left(1-\tau \right)\left(1-e^{-\lambda x}+\phi e^{-\lambda x}-\phi e^{-2\lambda x}\right)\right) \end{array}$$ (58)

Differentiating Eq 58 with the respective distribution parameters, to have

$$\begin{array}{c} \frac{\partial l}{\partial \mu }=n\;\text{ln}\;\sigma -n(\frac{\Gamma^{\prime }\left(\mu \right)}{\Gamma (\mu )}-\frac{\Gamma^{\prime }\left(\mu +\sigma \right)}{\Gamma (\mu +\sigma )})+\sum_{i=1}^{n}\;\text{ln}\;\left(1-e^{-\lambda x}+\lambda e^{-\lambda x}-\lambda e^{-2\lambda x}\right) \end{array}$$

$$\begin{array}{c} -\sum_{i=1}^{n}\;\text{ln}\;[1-\left(1-\tau \right)\left(1-e^{-\lambda x}+\phi e^{-\lambda x}-\phi e^{-2\lambda x}\right)] \end{array}$$ (59)

$$\begin{array}{c} \frac{\partial l}{\partial \sigma }=-n(\frac{\Gamma^{\prime }\left(\sigma \right)}{\Gamma (\sigma )}-\frac{\Gamma^{\prime }\left(\mu +\sigma \right)}{\Gamma (\mu +\sigma )})+\sum_{i=1}^{n}\;\text{ln}\;\left(e^{-\lambda x}-\lambda e^{-\lambda x}+\lambda e^{-2\lambda x}\right) \end{array}$$

$$\begin{array}{c} -\sum_{i=1}^{n}\;\text{ln}\;[1-\left(1-\tau \right)\left(1-e^{-\lambda x}+\phi e^{-\lambda x}-\phi e^{-2\lambda x}\right)] \end{array}$$ (60)

$$\begin{array}{c} \frac{\partial l}{\partial \phi }=\sum_{i=1}^{n}\frac{e^{-\lambda x}-1}{1-\phi +2\phi e^{-\lambda x}}+\left(\mu -1\right)\sum_{i=1}^{n}\frac{e^{-\lambda x}\left(1-e^{-\lambda x}\right)}{1-e^{-\lambda x}+\lambda e^{-\lambda x}-\lambda e^{-2\lambda x}}-\left(\sigma -1\right)\sum_{i=1}^{n}\frac{1-e^{-\lambda x}}{1-\phi +\phi e^{-\lambda x}} \end{array}$$

$$\begin{array}{c} +\left(\mu +\sigma \right)\sum_{i=1}^{n}\frac{e^{-\lambda x}\left(1-\tau \right)\left(1-e^{-\lambda x}\right)}{1-(\left(1-\tau \right)\left(1-e^{-\lambda x}\right)\left(1-\phi +2\phi e^{-\lambda x}\right))} \end{array}$$ (61)

$$\begin{array}{c} \frac{\partial l}{\partial \lambda }=-n\lambda -\sum_{i=1}^{n}\frac{2\phi \lambda e^{-\lambda x}}{1-\phi +2\phi e^{-\lambda x}}+\left(a-1\right)\sum_{i=1}^{n}\frac{\lambda e^{-\lambda x}\left(1-\phi +2\phi e^{-\lambda x}\right)}{1-e^{-\lambda x}+\phi e^{-\lambda x}-\lambda e^{-2\lambda x}} \end{array}$$

$$\begin{array}{c} -\left(\sigma -1\right)\sum_{i=1}^{n}\frac{1-\phi +2\phi e^{-\lambda x}}{1-\phi +\phi e^{-\lambda x}}+\left(\mu +\sigma \right)\sum_{i=1}^{n}\frac{\lambda e^{-\lambda x}\left(1-\tau \right)\left(1-\phi +2\phi e^{-\lambda x}\right)}{1-(\left(1-\tau \right)\left(1-e^{-\lambda x}\right)\left(1+\phi e^{-\lambda x}\right))} \end{array}$$ (62)

$$\begin{array}{c} \frac{\partial l}{\partial \tau }=\frac{n\mu }{\tau }\left(\mu +\sigma \right)\sum_{i=1}^{n}\frac{\left(1-e^{-\lambda x}\right)\left(1-\phi +2\phi e^{-\lambda x}\right)}{1-(\left(1-\tau \right)\left(1-e^{-\lambda x}\right)\left(1+\phi e^{-\lambda x}\right))} \end{array}$$ (63)

The maximum likelihood estimator of parameters can be obtained solving this nonlinear system of Eqs in 59, 60, 61, 62, 63. It is usually more convenient to use non-linear optimization algorithms such as quasi-Newton algorithm to numerically maximize the log-likelihood function.

### 5.8 Simulation study

In this section, a simulation study was performed using the MBTED in orfer to assess the performance of the maximum likelihood estimates of the distribution. To conduct this, 1000 samples of sizes 30,100,200 were generated from the quantile function of the MBTED for parameter values (2,3,2.5,-0.7,2),(3.2,1.3,1.5,0.5,0.5) and (3,3,3.5,0.2,2). The results of the simulation study are presented in Tables 1–3. These results show that the estimates for the mean is close to the parameter values as the sample sizes increase. Also, the mean square error decreases as the sample size increases.

**Table 1:** Simulation result of MBTED(2,3,2.5,-0.7,2).

Sample Size		*μ*	*σ*	*τ*	*ϕ*	λ
50	AE	2.342	3.533	1.142	2.107	2.470
	Bias	0.582	1.773	-0.617	0.346	0.710
	MSE	2.588	3.382	2.558	3.625	2.958
100	AE	2.160	4.043	0.915	2.508	2.298
	Bias	0.400	2.283	-0.844	0.748	0.538
	MSE	2.049	3.598	2.051	3.901	2.69
200	AE	2.120	4.646	0.999	2.965	1.902
	Bias	0.360	2.886	-0.760	1.205	0.142
	MSE	1.793	3.903	2.187	4.189	2.553

**Table 2:** Simulation result of MBTED(3.2,1.3,1.5,0.5,0.5).

Sample Size		*μ*	*σ*	*τ*	*ϕ*	λ
50	AE	2.695	1.339	0.463	0.553	1.319
	Bias	1.295	-0.060	-0.936	-0.846	-0.080
	MSE	7.798	1.626	2.481	1.898	1.462
100	AE	2.318	1.204	0.364	0.681	1.191
	Bias	0.918	-0.195	-1.035	-0.718	-0.208
	MSE	5.666	1.493	1.808	1.301	1.200
200	AE	1.990	1.171	0.314	0.717	1.090
	Bias	0.590	-0.228	-1.085	-0.682	-0.309
	MSE	1.901	1.262	1.536	1.279	1.163

**Table 3:** Simulation result of MBTED(3,3,3.5,0.2,2).

Sample Size		*μ*	*σ*	*τ*	*ϕ*	λ
50	AE	2.556	4.762	2.437	1.199	5.135
	Bias	0.216	2.422	0.097	-0.846	2.795
	MSE	2.714	5.304	3.402	5.973	6.176
100	AE	2.594	4.446	2.409	1.200	4.503
	Bias	0.254	2.106	0.069	-1.140	2.163
	MSE	2.377	4.611	3.320	5.717	4.616
200	AE	2.366	4.037	2.489	0.978	4.295
	Bias	0.026	1.697	0.149	-1.361	1.955
	MSE	2.186	3.104	3.086	3.442	4.437

### 5.9 Application to real data

In this section, applications to two real data(Medicine and Behavioral datasets) are presented to illustrate the importance and the fit of the MBTED. The maximum likelihood estimates (M.L.E) of the distribution and that of the competitive distributions will be obtained. The goodness of fit of the distributins was assessed using the log-likelihood, Akaike’s information criterion (AIC), Bayesian information criterion (BIC), corrected Akaike’s information criterion (CAIC), Hannan-Quinn Information Criterion(HQIC) and the Kolmogorov Smirnov test for the models. The fits of the MBTED is compared with other competitive distributions which are Exponentiated Generalized Weibull(EGW) [19], Exponentiated Kumuraswamy Exponential(EKE) [20], Beta Burr XII [21], Modified Beta Gompertz(MBG) [22], Exponential, Exponentiated Transmuted Exponential(ETED) [15]. The p.d.fs of these distributions are as follows:

$$\begin{array}{c} EGW=\alpha \beta \left(\tau \gamma^{\tau }x^{\tau -1}e^{-{\left(\gamma x\right)}^{\tau }}\right){\left(1-e^{-{\left(\gamma x\right)}^{\tau }}\right)}^{\alpha -1}{\left[1-{\left(1-e^{-{\left(\gamma x\right)}^{\tau }}\right)}^{\alpha }\right]}^{\beta -1} \end{array}$$

$$\begin{array}{c} EKE=\alpha \beta \gamma \left(\tau e^{-\tau x}\right){\left(1-e^{-\tau x}\right)}^{\alpha -1}{\left({\left(e^{-\tau x}\right)}^{\alpha }\right)}^{\beta -1}{\left(1-{\left(1-{\left(1-e^{-\tau x}\right)}^{\alpha }\right)}^{\beta }\right)}^{\left(\gamma -1\right)} \end{array}$$

$$\begin{array}{c} BBXII=\frac{\left(\gamma \tau x^{\tau -1}{\left(1+x^{\tau }\right)}^{-\gamma -1}\right){\left(1-{\left(\frac{1}{1+x^{\theta }}\right)}^{\tau }\right)}^{\alpha -1}{\left({\left(\frac{1}{1+x^{\theta }}\right)}^{\tau }\right)}^{\beta -1}}{B(\alpha ,\beta )} \end{array}$$

$$\begin{array}{c} MBG=\frac{\gamma^{\alpha }{\left(1-e^{-\frac{\tau }{\theta }\left(e^{\theta x}\right)-1}\right)}^{\alpha -1}{\left(e^{-\frac{\tau }{\theta }\left(e^{\theta x}\right)-1}\right)}^{\beta -1}\left(\tau e^{\theta x-\frac{\tau }{\theta }\left(e^{\theta x}-1\right)}\right)}{B\left(\alpha ,\beta \right){\left[1-\left(\left(1-c\right)\left(1-e^{-\frac{\tau }{\theta }\left(e^{\theta x}\right)-1}\right)\right)\right]}^{\left(\alpha +\beta \right)}} \end{array}$$

$$\begin{array}{c} ED=\lambda e^{-\lambda x} \end{array}$$

#### 5.9.1 Survival times of breast cancer patients

The real data set represent the survival times of 121 patients with breast cancer obtained from a large hospital in a period from 1929 to 1938 [23]. The data are: 0.3, 0.3, 4.0, 5.0, 5.6, 6.2, 6.3, 6.6, 6.8, 7.4, 7.5, 8.4, 8.4, 10.3, 11.0, 11.8, 12.2, 12.3, 13.5, 14.4, 14.4, 14.8, 15.5, 15.7, 16.2, 16.3, 16.5, 16.8, 17.2, 17.3, 17.5, 17.9, 19.8, 20.4, 20.9, 21.0, 21.0, 21.1, 23.0, 23.4, 23.6, 24.0, 24.0, 27.9, 28.2, 29.1, 30.0, 31.0, 31.0, 32.0, 35.0, 35.0, 37.0, 37.0, 37.0, 38.0, 38.0, 38.0, 39.0, 39.0, 40.0, 40.0, 40.0, 41.0, 41.0, 41.0, 42.0, 43.0, 43.0, 43.0, 44.0, 45.0, 45.0, 46.0, 46.0, 47.0, 48.0, 49.0, 51.0, 51.0, 51.0, 52.0, 54.0, 55.0, 56.0, 57.0, 58.0, 59.0, 60.0, 60.0, 60.0, 61.0, 62.0, 65.0, 65.0, 67.0, 67.0, 68.0, 69.0, 78.0, 80.0, 83.0, 88.0, 89.0, 90.0, 93.0, 96.0, 103.0, 105.0, 109.0, 109.0, 111.0, 115.0, 117.0, 125.0, 126.0, 127.0, 129.0, 129.0, 139.0, 154.0.

**Fig 7:**
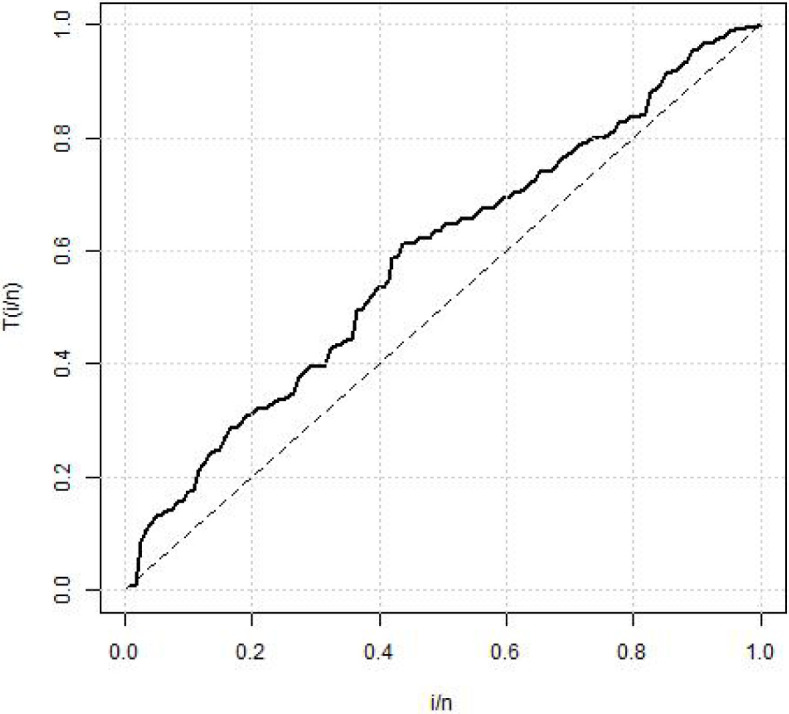
TTT plot of survival times of breast cancer.

**Fig 8:**
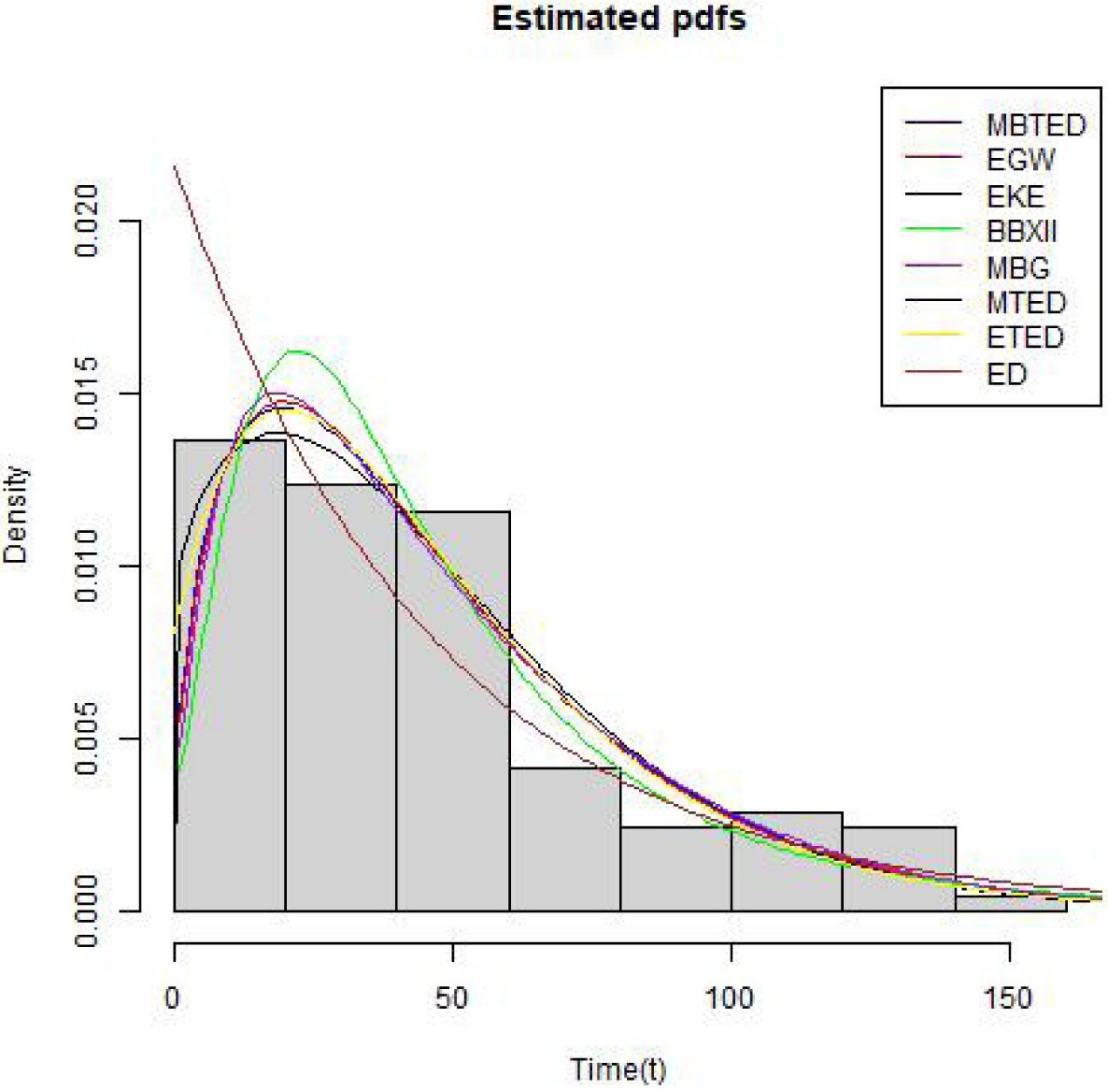
Estimated pdf plots.

Table 4 shows the summary statistics for the real data. Fig 7 is the TTT plots of the dataset which shows a non decreasing curve. Fig 8 shows the fitted plot of the data using the MBTED and the competitive distributions. This indicated that the model fits the data. Table 5 reveals that the modified beta transmuted exponential distribution gives the best fit when compared to its submodels, due to lowest values of AIC, BIC, CAIC and HQIC therefore making it the preferred model to consider for this data.

**Table 4:** Table displaying descriptive analysis of survival time of breast cancer patients.

Minimum	First Quartile	Median	Mean	Third Quartile	Maximum
0.30	17.50	40.00	46.33	60.00	154.00

**Table 5:** Table displaying results of analysis of survival times of breast cancer patients.

Model	Parameter	Estimate	L	AIC	CAIC	BIC	HQIC	KS(p-value)
MBTED	*ϕ*	0.693	578.30	1167.095	1167.617	1181.074	1172.773	0.048(0.936)
	*μ*	4.436						
	*σ*	0.6050						
	*τ*	-0.971						
	λ	0.011						
ETED	λ	0.019	581.8153	1169.632	1169.843	1183.23	1175.02	0.055(0.079)
	*ϕ*	0.663						
	*σ*	1.645						
EGW	*α*	1.258	579.603	1169.207	1169.729	1183.186	1174.884	0.058(0.803)
	*β*	1.351						
	*γ*	1.152						
	*τ*	52.384						
EKE	*α*	2.419	579.772	1169.545	1170.067	1183.524	1175.223	0.061(0.756)
	*β*	1.384						
	*γ*	0.689						
	*τ*	0.022						
	*θ*							
BBXII	*α*	61.620	582.383	1174.764	1175.285	1188.743	1180.441	0.774(0.462)
	*β*	27.297						
	*γ*	0.372						
	*τ*	0.792						
MBG	*α*	2.756	579.43	1170.864	1171.601	1187.639	1177.677	0.061(0.761)
	*β*	0.679						
	*γ*	2.090						
	*τ*	0.003						
	*θ*	0.030						
ED	λ	0.022	585.1277	1172.26	1175.05	1172.29	1173.391	0.120(0.059)

#### 5.9.2 Recidivism failure time data

**Fig 9:**
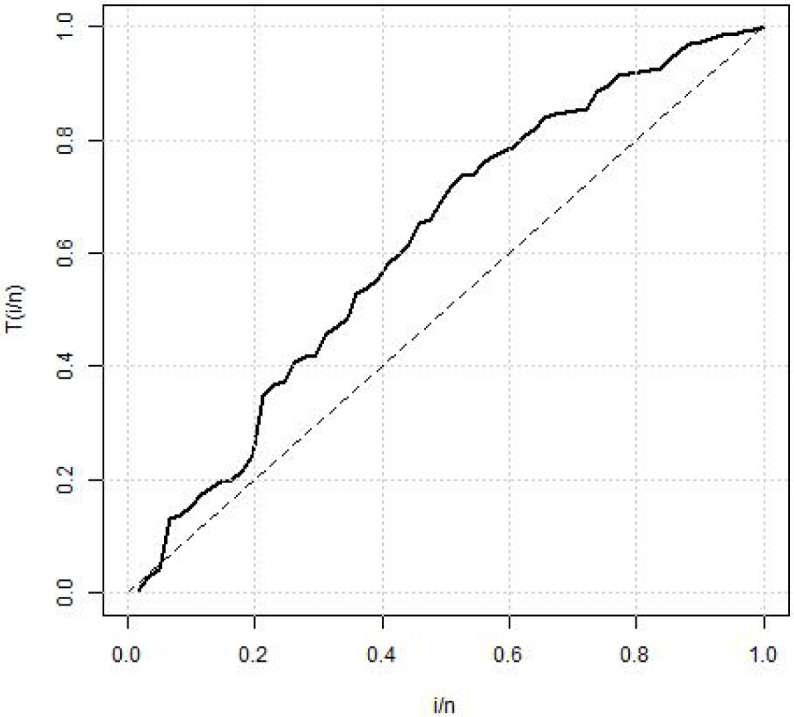
TTT plot of recidivism failure times data.

**Fig 10:**
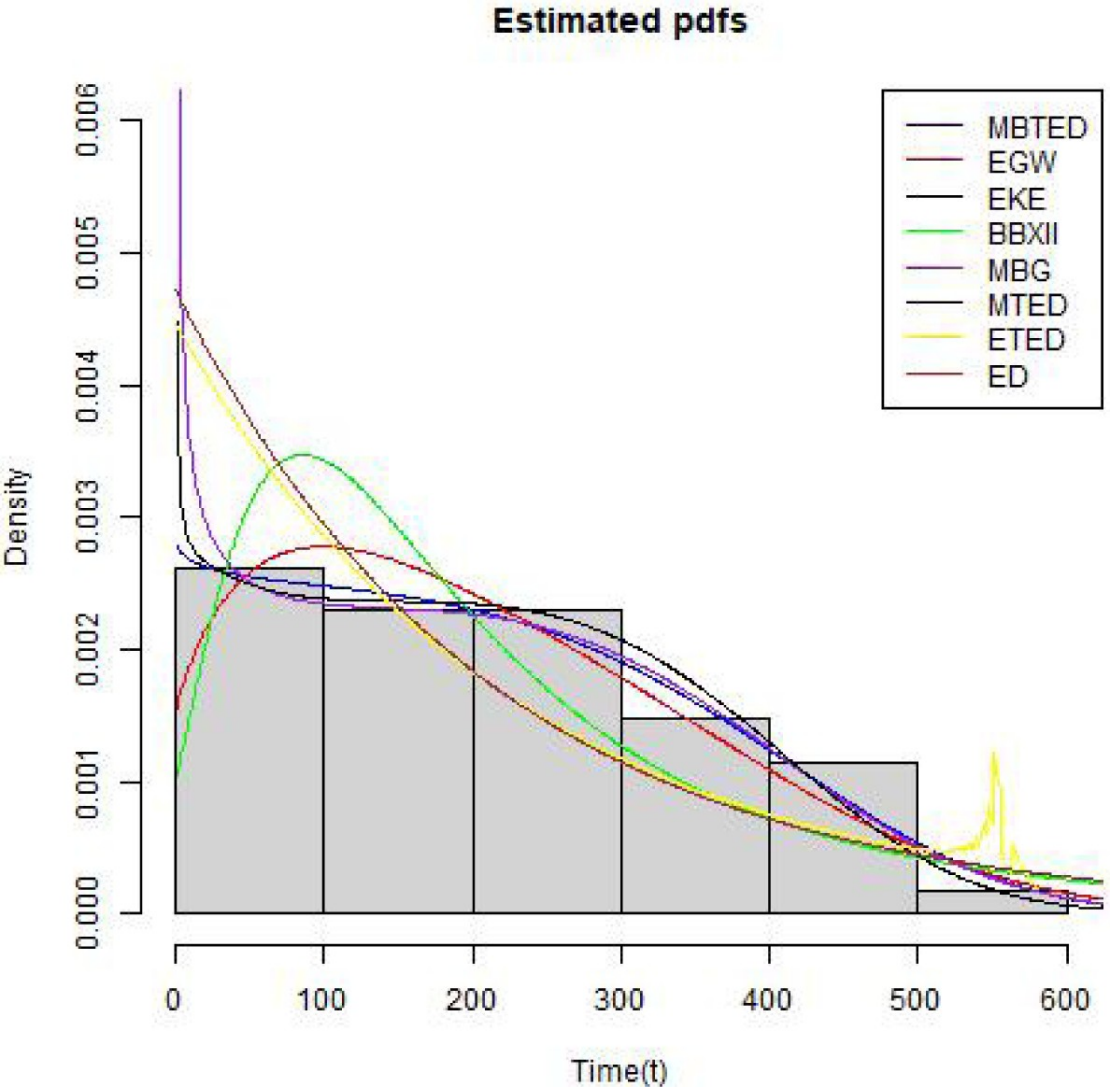
Estimated pdf plots.

The second data consists of 61 observed recidivism failure times (in days) revealed by correctional institutions in Columbia USA by [24]. The failure times data were:138, 141, 146, 217, 217, 228, 156, 162, 168, 183, 185, 1, 6, 9, 29, 30, 34, 39, 422, 438, 441, 465, 41, 44, 45, 49, 56, 84, 89, 91, 100, 103, 104, 238, 241, 252, 258, 271, 275, 276, 279, 282, 305, 313, 329, 331, 334, 336, 336, 362, 209, 233, 384, 404, 408, 115, 119, 124, 198, 486, 556. Table 6 shows the summary statistics for the real data. Fig 9 is the TTT plots of the dataset which shows a non decreasing curve. Fig 10 shows the fitted plot of the data using the MBTED and the competitive distributions. This indicated that the model fits the data. Table 7 reveals that the modified beta transmuted exponential distribution gives the best fit when compared to its submodels, due to lowest values of AIC, BIC, CAIC and HQIC therefore making it the preferred model to consider for this data. Clearly, based on the values of the criteria used, all of the two applications provided indicate that the MBTED distribution is superior to the other models. It has lower values for the LL, AIC, CAIC, BIC, and HQIC than it does for the others.

**Table 6:** Table displaying descriptive analysis of recidivism failure time data.

Minimum	First Quartile	Median	Mean	Third Quartile	Maximum
1.0	100.0	209.0	211.7	313.0	556.00

**Table 7:** Table displaying results of analysis of survival times of breast cancer patients.

Model	Parameter	Estimate	L	AIC	CAIC	BIC	HQIC	KS(p-value)
MBTED	*ϕ*	0.567	379.517	769.034	770.125	779.589	773.171	0.044(0.999)
	*μ*	148.429						
	*σ*	0.002						
	*τ*	-0.907						
	λ	0.006						
ETED	λ	0.079	387.658	781.316	781.737	787.649	783.798	0.136(0.208)
	*ϕ*	-0.008						
	*σ*	0.059						
EGW	*α*	0.226	380.684	771.367	772.458	781.922	775.504	0.049(0.998)
	*β*	0.273						
	*γ*	3.532						
	*τ*	266.704						
EKE	*α*	13.158	382.232	774.463	775.5539	785.0174	778.5994	0.062(0.971)
	*β*	7.250						
	*γ*	0.120						
	*τ*	0.003						
BBXII	*α*	78.35	388.614	787.228	788.319	797.783	791.365	0.109(0.456)
	*β*	36.520						
	*γ*	0.286						
	*τ*	0.736						
MBG	*α*	0.741	379.576	771.153	772.709	783.818	776.117	0.051(0.996)
	*β*	1.188						
	*γ*	0.454						
	*τ*	0.003						
	*θ*	0.002						
ED	λ	0.005	387.671	777.343	777.410	779.453	778.170	0.136(0.208)

## 6 Conclusion

In this article, a new family distribution called the Modified Beta Transmuted-G family is introduced. The properties of the family such as moments, generating functions, quantile function, random number generation, reliability function and order statistics were extensively studied. Furthermore, expressions for the the maximum likelihood estimation of parameters for the Modified Beta Transmuted-G family of distribution were derived. An exponential distribution was applied as a baseline distribution for the modified beta transmuted-G to derive the modified beta transmuted exponential distribution. The properties of the modified beta transmuted exponential distribution were also been discussed and estimation of parameters done using the maximum likelihood estimation method. The modified beta transmuted exponential distribution was applied on a real data set in which it was observed that the modified beta transmuted exponential distribution provides better fit than its submodels. We anticipate that the proposed model will be used to investigate a wider range of applications in diverse areas of applied research in the future, and that it will be considered a superior alternative to the baseline model. The model could also be applied in other fields such as machine learning and artificial intelligence.

## Supporting information

S1 Dataset (DOCX)

## References

[1] Eugene N, Lee C, Famoye F. *β*-Normal Distribution and its Applications. Communications in Statistics—Theory and Methods, 2002; 31(4), 497–512. doi: 10.1081/STA-120003130

[2] Bourguignon M, Silva R, GM C. The Weibull-G family of Probability Distributions. Journal of Data Science, 2014; 12, 53–68

[3] Yousof H, Rasekhi M, Afify A, Ghosh I, Alizadeh M, Hamedani G. The Beta Weibull-G Family of Distributions: Theory, Characterizations and Applications. Pakistan Journal of Statistics, 2017; 33, 95–116

[4] Nadarajah S, Teimouri M, Shih SH. Modified Beta Distributions. Sankhya Ser. B, 2014; 76, 19–48. doi: 10.1007/s13571-013-0077-0

[5] Rahman MM, Al-Zahrani B, Shahbaz MQ. A general transmuted family of distributions. Pakistan Journal of Statistical Operation Research, 2018; 14, 451–469 doi: 10.18187/pjsor.v14i2.2334

[6] Alizadeh M, Cordeiro GM, Pinho LG, Ghosh I. The Gompertz-G family of distributions. Journal of Statistical Theory and Practice, 2017; 11:1, 179–207. doi: 10.1080/15598608.2016.1267668

[7] Silva FG, Percontini A, de Brito E, Ramos MW, Venancio R, Cordeiro GM. The odd Lindley-G family of distributions. Austrian J Stat, 2017; 46,65–87 doi: 10.17713/ajs.v46i1.222

[8] Shaw WT, Buckley IR. The Alchemy of Probability Distributions: Beyond Gram-Charlier Expansions, and a Skew-Kurtotic-Normal Distribution from a Rank Transmutation Map., 2009; 0901.0434

[9] Afify A, Yousof HM, Nadarajah S. The Beta Transmuted-H Family for Lifetime Data. Statistics and Its Interface, 2017; 10, 505–520. doi: 10.4310/SII.2017.v10.n3.a13

[10] Merovci F, Alizadeh M, Yousof HM, Hamedani GG. The exponentiated transmuted-G family of distributions: Theory and applications. Communications in Statistics—Theory and Methods, 2017; 46:21, 10800–10822 doi: 10.1080/03610926.2016.1248782

[11] Moniem AIB, Seham M. Transmuted Gompertz Distribution. Comput. Appl. Math., 2005; 1, 88–96

[12] Jafari AA, Tahmasebi S Alizadeh M. The beta-Gompertz distribution. Revista Colombiana de Estadistica, 2014; 37, 141–158 doi: 10.15446/rce.v37n1.44363

[13] Owoloko EA, Oguntunde PE, Adejumo AO. Performance rating of the transmuted exponential distribution: an analytical approach. SpringerPlus, 2015; 4, 818. doi: 10.1186/s40064-015-1590-6 26722638 PMC4690830

[14] Nadarajah S, Kotz S. The beta exponential distribution. Reliab. Eng. Syst. Saf., 2006; 91, 689–697 doi: 10.1016/j.ress.2005.05.008

[15] Kareema AA, Ashraf AM. Exponentiated Transmuted Exponential Distribution. Journal of University of Babylon, 2018; 26:78–90.

[16] Merovci F. Transmuted Lindley Distribution. International Journal of Open Problems in Computer Science and Mathematics, 2013; 6(2):63–72

[17] Merovci F and Sharma VK. The Beta-Lindley Distribution: Properties and Applications. Journal of Applied Mathematics, 2014, 1–10 doi: 10.1155/2014/198951

[18] Okereke EW. Exponentiated transmuted lindley distribution with applications. Open Journal of Mathematical analysis, 2019; 3, 1–18. doi: 10.30538/psrp-oma2019.0035

[19] Oguntunde P, Odetunmibi O, Adejumo AO. On the Exponentiated Generalized Weibull Distribution: A Generalization of the Weibull Distribution. Indian Journal of Science and Technology, 2015. doi: 10.17485/ijst/2015/v8i35/67611

[20] Rodrigues JA, Silva ACM. The Exponentiated Kumaraswamy-Exponential Distribution. British Journal of Applied Science & Technology, 2005; 10, 1–12 doi: 10.9734/BJAST/2015/16935

[21] Paranaíba PF, Ortega EMM, Cordeiro GM, Pescim RR. The beta Burr XII distribution with application to lifetime data. Computational Statistics & Data Analysis, 2011; 55, 1118–1136, ISSN 0167-9473. doi: 10.1016/j.csda.2010.09.009

[22] Elbatal I, Jamal F, Chesneau C, Elgarhy M, Alrajhi S. The Modified Beta Gompertz Distribution: Theory and Applications. Mathematics. 2019; 7(1):3. doi: 10.3390/math7010003

[23] Lee. Statistical methods for survival data analysis. John Wiley. 1992

[24] Stollmack S, Harris CM. Failure-Rate Analysis Applied to Recidivism Data. Operation Research, 1974; 22(6),1139–1282 doi: 10.1287/opre.22.6.1192

