# Supplementary material for: The modified beta transmuted family of distributions with applications using the exponential distribution
Source: PLoS One. 2021 Nov 18;16(11):e0258512. doi: 10.1371/journal.pone.0258512 (PMC8601563; doi:10.1371/journal.pone.0258512)
Supplement: S1 Dataset — (DOCX) [file pone.0258512.s001.docx]

**Dataset used for The Modified Beta Transmuted Family of Distributions with**

**applications using the Exponential Distribution**

The real data set represent the survival times of 121 patients with breast cancer

obtained from a large hospital in a period from 1929 to 1938 [6]. The data are: 0.3, 0.3,

4.0, 5.0, 5.6, 6.2, 6.3, 6.6, 6.8, 7.4, 7.5, 8.4, 8.4, 10.3,11.0, 11.8, 12.2, 12.3, 13.5, 14.4,

14.4, 14.8, 15.5, 15.7, 16.2, 16.3, 16.5, 16.8, 17.2, 17.3, 17.5,17.9, 19.8, 20.4, 20.9, 21.0,

21.0, 21.1, 23.0, 23.4, 23.6, 24.0, 24.0, 27.9, 28.2, 29.1, 30.0, 31.0,31.0, 32.0, 35.0, 35.0,

37.0, 37.0, 37.0, 38.0, 38.0, 38.0, 39.0, 39.0, 40.0, 40.0, 40.0, 41.0, 41.0,41.0, 42.0, 43.0,

43.0, 43.0, 44.0, 45.0, 45.0, 46.0, 46.0, 47.0, 48.0, 49.0, 51.0, 51.0, 51.0, 52.0,54.0, 55.0,

56.0, 57.0, 58.0, 59.0, 60.0, 60.0, 60.0, 61.0, 62.0, 65.0, 65.0, 67.0, 67.0, 68.0, 69.0,78.0,

80.0,83.0, 88.0, 89.0, 90.0, 93.0, 96.0, 103.0, 105.0, 109.0, 109.0, 111.0, 115.0, 117.0,

125.0,126.0, 127.0, 129.0, 129.0, 139.0, 154.0.
